# Material challenges for solar cells in the twenty-first century: directions in emerging technologies

**DOI:** 10.1080/14686996.2018.1433439

**Published:** 2018-04-10

**Authors:** Samy Almosni, Amaury Delamarre, Zacharie Jehl, Daniel Suchet, Ludmila Cojocaru, Maxime Giteau, Benoit Behaghel, Anatole Julian, Camille Ibrahim, Léa Tatry, Haibin Wang, Takaya Kubo, Satoshi Uchida, Hiroshi Segawa, Naoya Miyashita, Ryo Tamaki, Yasushi Shoji, Katsuhisa Yoshida, Nazmul Ahsan, Kentaro Watanabe, Tomoyuki Inoue, Masakazu Sugiyama, Yoshiaki Nakano, Tomofumi Hamamura, Thierry Toupance, Céline Olivier, Sylvain Chambon, Laurence Vignau, Camille Geffroy, Eric Cloutet, Georges Hadziioannou, Nicolas Cavassilas, Pierre Rale, Andrea Cattoni, Stéphane Collin, François Gibelli, Myriam Paire, Laurent Lombez, Damien Aureau, Muriel Bouttemy, Arnaud Etcheberry, Yoshitaka Okada, Jean-François Guillemoles

**Affiliations:** a NextPV, LIA RCAST-CNRS, The University of Tokyo, Tokyo, Japan; b Research Center for Advanced Science and Technology, The University of Tokyo, Tokyo, Japan; c Okadalab, Research Center for Advanced Science and Technology (RCAST), The University of Tokyo, Tokyo, Japan; d IPVF, UMR CNRS 9006, Palaiseau, France; e Centre for Nanoscience and Nanotechnology (C2N), CNRS, University Paris-Sud/Paris-Saclay, Palaiseau, France; f Komaba Organization for Educational Excellence, Faculty of Arts and Sciences, The University of Tokyo, Tokyo, Japan; g Department of General Systems Studies, Graduate School of Arts and Sciences, The University of Tokyo, Tokyo, Japan; h University of Bordeaux, Institut des Sciences Moléculaires (ISM), CNRS (UMR 5255), Talence Cédex, France; i University of Bordeaux, IMS, CNRS UMR 5218, Talence, France; j Université de Bordeaux, Laboratoire de Chimie des Polymères Organiques (LCPO), UMR 5629, ENSCBP, IPB, Pessac Cedex, France; k Aix Marseille Université, CNRS, Université de Toulon, IM2NP UMR 7334, Marseille, France; l Institut Lavoisier de Versailles (ILV), Université de Versailles Saint-Quentin (UVSQ), Université Paris-Saclay, Versailles, France

**Keywords:** Photovoltaics, semiconductors, nanotechnologies, energy conversion, efficiency, luminescence, devices, 50 Energy Materials, 206 Energy conversion / transport / storage / recovery, 209 Solar cell / Photovoltaics, 502 Electron spectroscopy, 505 Optical / Molecular spectroscopy

## Abstract

Photovoltaic generation has stepped up within the last decade from outsider status to one of the important contributors of the ongoing energy transition, with about 1.7% of world electricity provided by solar cells. Progress in materials and production processes has played an important part in this development. Yet, there are many challenges before photovoltaics could provide clean, abundant, and cheap energy. Here, we review this research direction, with a focus on the results obtained within a Japan–French cooperation program, NextPV, working on promising solar cell technologies. The cooperation was focused on efficient photovoltaic devices, such as multijunction, ultrathin, intermediate band, and hot-carrier solar cells, and on printable solar cell materials such as colloidal quantum dots.

## Graphical abstract

**Figure:**
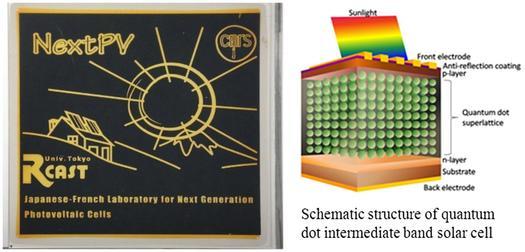

## 1. Introduction

Material research has gone a long way since the photovoltaic effect was discovered by Becquerel, in 1839 [1]. It is only with the discovery of the photosensitivity of selenium, the first technological semiconductor, and the fabrication of wafers with about 1% conversion efficiency the perspective of large-scale applications was first mentioned [2].

In 1954, the first silicon ‘photocell for converting solar radiation into electrical power’ was reported, with an efficiency of 6% [3] and triggered the development of the technology, as this performance turned the photovoltaic technology into one of the best contenders for applications to power the nascent satellite industry.

### 1.1. Achievements of mature technologies

Within the last 60 years, in a context of depletion of oil deposits and increasing pressure from global warming, solar cells have emerged to offer a credible alternative to fossil fuels, providing large amounts of renewable energy at affordable prices. PV systems already provide 1.7% of the gross electricity production in Organisation for Economic Co-operation and Development [4], while their contribution was negligible (below 0.01%) in 1990. According to International Energy Agency, photovoltaics (PV) is the energy technology with the fastest growth and should pass the 300-GW global installation in 2017. This rapid evolution illustrates the complementarity of fundamental research, dedicated to reaching highest performances and industrial developments, turning laboratory results into commercial systems.

It is customary to distinguish three generations of solar cells

(1) Silicon-based solar cells (mono- and poly-crystalline silicon) constituted the first PV sector to emerge, taking advantage of the processing feedback and supply feedstock provided by the microelectronics industry [5]. Silicon-based solar cells cover over 80% of the world installed capacity today [6,7] and currently represent 90% of the market shares [8].
(2) Thin film solar cells based on CdTe, copper indium gallium selenide (CIGS), or amorphous silicon were developed as a cheaper alternative to crystalline silicon cells. They provide better mechanical properties, allowing for flexible usages at the risk of a lower efficiency. While the first generation of solar cell was essentially a case for microelectronics, the development of thin-films involved new growth methods and opened the sector to other fields, such as electrochemistry.
(3) ‘Third generation’ solar cells (tandem, perovskite, dye-sensitized, organic, new concepts, …) account for a broad spectrum of concepts, ranging from low-cost low-efficiency systems (dye-sensitized, organic solar cells) to high-cost high-efficiency systems (III–V multijunction), with various purposes from building integration to space applications. Third-generation solar cells are sometimes referred to as ‘emerging concepts’ because of their low market penetration, although some of them have been investigated for over 25 years.

The ability of any solar technology to address the issue of energy production relies on the balance between the investments required to produce and install a module (i.e. several cells assembled in an operable device), and the total energy provided by the module, which in turn depends on its lifetime and conversion efficiency. Research and development in solar technologies has led to remarkable improvements on all aspects, which we will briefly present to account for the situation of the field. This review will focus on the achievements of the mature first- and second-generation technologies to introduce the challenges currently faced by new concepts.

### 1.2. Production costs

The more developed a technology is, the less expensive it becomes to increase the installed capacity by an additional watt [9]. With the largest production by far, silicon PV cells exhibits an impressive reduction of the production expenses over the last decades. Recent studies [9] estimate indeed that each doubling of the installed capacity of mono- or polycrystalline-based photovoltaic systems leads to a reduction of 12% in the cumulative energy demand, and therefore a reduction of the selling price by 20% and of the greenhouse gases by 20%. As a result, while one watt of peak power amounted to a production cost of 100 USD, 100 MJ, and 10 kg CO_2_ in 1975, it is now estimated to 0.5 USD, 15 MJ, and 0.8 kg CO_2_ [9,10] corresponding to an average energy payback time of about 2 years [11] under an insolation of 1700 kWh/m^2^/yr.

In comparison with silicon-based solar cells, thin-film systems require less material per surface, avoid costly purification steps, and do not necessitate silver electric contacts. As a result, despite a much smaller volume produced, thin-film systems compare favorably with silicon-based solar cells in terms of embedded energy and energy payback time [12], as well as in terms of cost.

### 1.3. Lifetime

This payback duration is to be compared to the lifetime of the module, which is notably limited by deteriorations induced by heat and humidity. Despite initial concerns, thin-film systems appear to have lifetime comparable to that of silicon panels, albeit generally lower. A typical degradation rate for the efficiency of both technologies is estimated to −0.5–−1% per year [13], corresponding to a lifetime of 25–40 years before the nominal efficiency drops by 20%. This large lifespan enables solar technologies to produce over their period of use around 14–20 times the energy invested in the production of the device [11,12].

### 1.4. Efficiency

The most symbolic indicator used to assess and compare PV technologies is certainly the conversion efficiency, expressing the ratio between solar energy input and electrical energy output. Efficiency aggregates many constituting parameters of the system, such as the short-circuit current, the open-circuit voltage, and the fill factor, which in turn depend on the fundamental properties of the material as well as the manufacturing defects.

For commercially available technologies, an upper bound for the efficiency is set by the celebrated Shockley–Queisser (SQ) limit [14], which accounts for the balance between photogeneration and radiative recombination of thermalized carriers. Any optical, conversion, or electrical loss would result in an efficiency lower than predicted by the SQ formula [15]. In addition to the defects affecting individual solar cells, the engineering required to mount solar cells into a solar module can induce further deficit in the efficiency.

**Figure 1.**
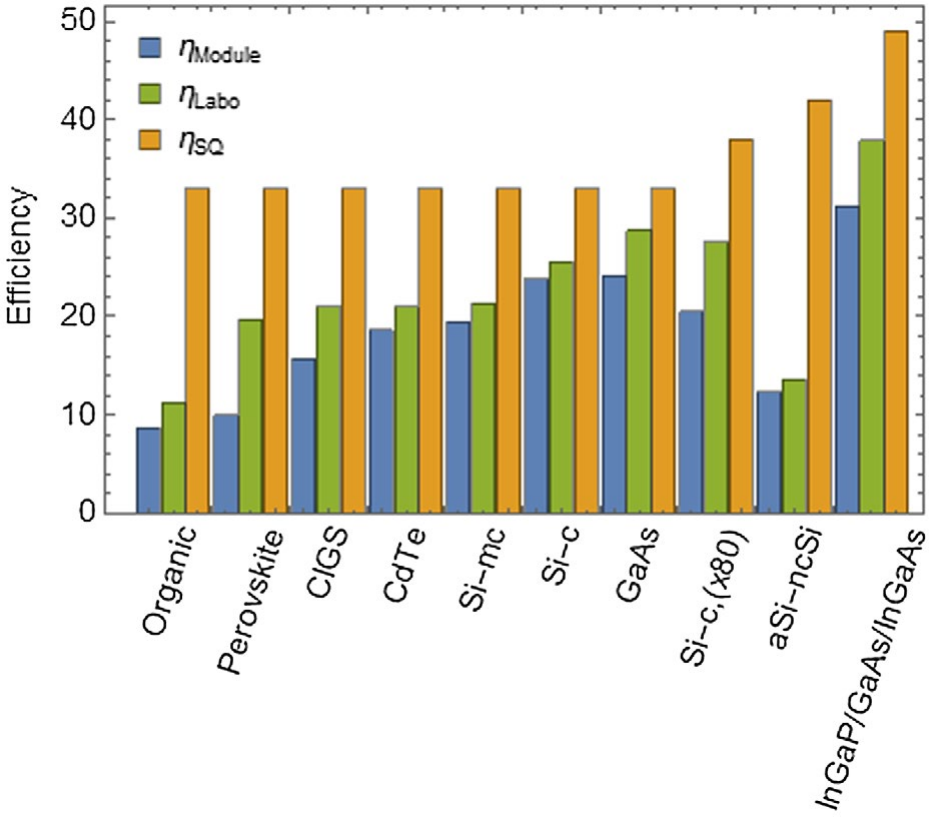
Best module (blue), best cell (green), and Shockley–Queisser (SQ) efficiency (yellow) of major technologies. Si-mc, Si-c, Si-c(x80), and aSi-ncSi stand for multicrystalline, crystalline, crystalline under 80 suns illumination and amorphous/nanocrystalline silicon, respectively. Data from [16,18].

The efficiencies of the best module and cell of each major technology are compared to the corresponding SQ limit in Figure 1, using data from [16]. The discrepancy between the best cell efficiency and the upper SQ limit gives a picture of the room for improvement left for fundamental research, while difference between the best cell and the best module indicates how much could be gained by improving the transfer of laboratory prototypes into integrated systems. It appears that most mature technologies are not limited by integration losses (*η*_module_ ≃ *η*_cell_), while perovskite systems exhibit the larger shortfall $\eta_{\text{module}}=0.5\;\eta_{\text{cell}}$. Mature technologies already approach the SQ limit, but most of them show little evolution over the last five years [17,18].

**Figure 2.**
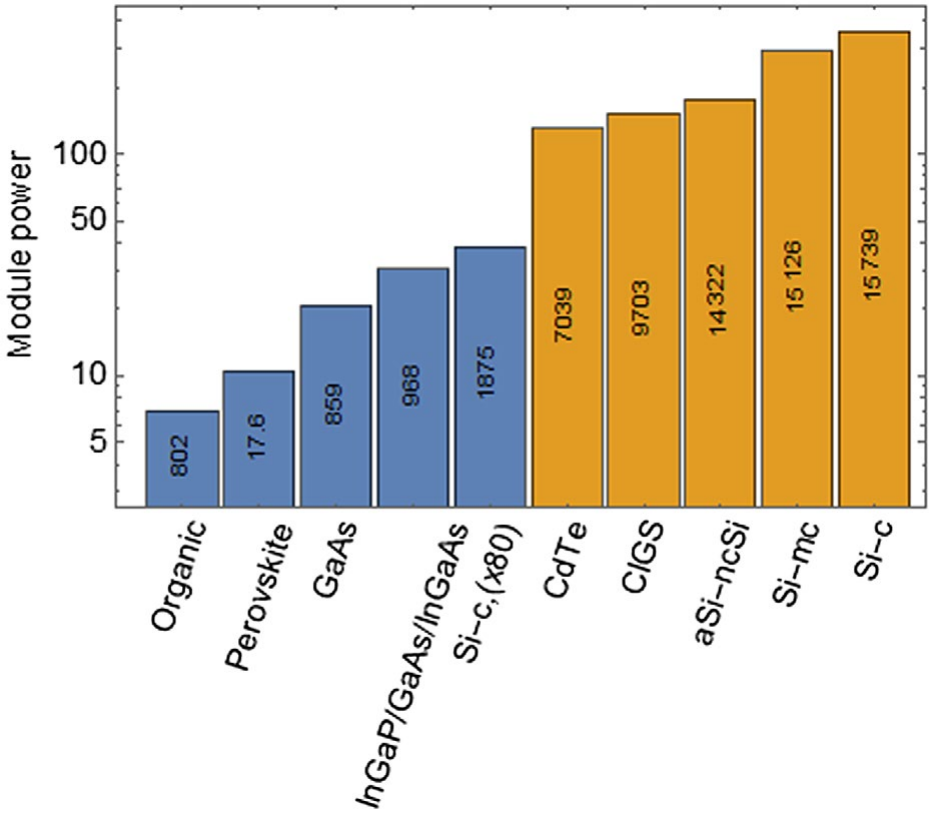
Output power of the best module for each technology. For each technology, the surface of the best module is indicated in cm^2^. Mature technologies (1st and 2nd generations) are depicted in yellow, 3rd generation in blue. Data from [16,18].

While the efficiency holds information on the overall quality of the materials, it does not indicate whether the material at stake can be readily expanded to larger surfaces. By contrast with small samples, which can be extracted from carefully selected defectless regions of a larger plate, this up-scaling requires indeed an excellent control of the homogeneity of the growth processes. In addition to the efficiency analysis, a complementary approach is therefore used to compare the power output delivered by actual modules – or equivalently the product of the surface of the module by its efficiency. The result, displayed in Figure 2, draws a clear line between industrially mature technologies, upscaled to large surface and hence able to able to deliver more than 100 W, and emergent technologies, which are for the time being limited to smaller surfaces.

Finally, a last indicator is the real amount of kWh produced in operation as compared to the nominal power of the modules (measured under 1000 W incident power and at 25 °C). Here, Si technologies are handicapped by the more rapid decrease in efficiency of Si solar cells when either temperature increases (operation temperatures are in the range of 60–80 °C) or illumination intensity decreases, as compared to competing technologies.

### 1.5. Material challenges for the twenty-first century

We have presented the state of the art of photovoltaics, and the impressive progress made in the previous decades. At about 300-gigawatt peak installed and likely very soon 2% of the electric energy production, there are still significant challenges before photovoltaic energy can be a significant fraction of the overall energy production:

(i) Efficiencies are still significantly below those allowed by thermodynamics. As explained below, photovoltaic devices have a high theoretical energy conversion limit: above 33% for single junctions, and ultimately close to 90% if suitable materials can be found. Especially, relevant are materials for tandems, and for new conversion processes such as intermediate band or hot-carrier solar cells. Efficiencies are highly dependent on the quality of the materials and extremely sensitive to chemical and structural defects, even at low concentration.
(ii) Materials availability, and processability to achieve low cost. Extremely low cost could be achieved if highly scalable and low-cost processes (e.g. printing technologies) could be used to produce high-quality materials. Materials availability was also seen to enter the equation in the past decade, as the growth of the production causes concerns about the long-term sustainability of the technology.
(iii) Durability and material aging at solar cell and module level are also an issue as this affects the reliability of the technology and also ultimately the cost. This concerns a lot structure materials and encapsulation, but intrinsic stability of the active materials was often found to be an issue to be solved first, and caused the failure of some technologies in the past, as e.g. Cu_2_S/CdS.
(iv) Life cycle constraints (toxicity, recyclability, including structure materials) may become prevalent as the productions reaching volumes (terrawatt scale) where concerns on supply chain, including environmental ones come to the front. An increasing number of researches are oriented toward addressing these issues.
(v) Finally, integration to the global energy system (system, storage) and to the built environment (storage, aspect) are becoming hot topics as the penetration in the energy production (2% of electricity) is getting close to the point where power management is critical, and synergies with power electronics and electrochemical storage are considered. Again, with both increased performance and better affordability, a larger number of applications appear. They often require (e.g. architectural integration) that other properties (e.g. esthetics) are considered.

Most of the challenges listed above will likely require radically new pathways. While today, performances (including efficiency and reliability) and competitiveness have been sufficient to reach a visible penetration in the energy production, a significant contribution to this mix will require new levels of performance. Performance does not only have an impact on cost, it also contributes positively to sustainability. At the terrawatt level, environmental footprint and life cycle issue will grow in importance as well. For these reasons we have chosen to focus in this issue on emerging approaches that may help solve these issues. We will consider first how the efficiency frontier could be approached. We will then discuss new materials for solar competitiveness and improved integration, especially molecular, colloidal or hybrid materials. We will finally give some examples on how material or device characterization, and modeling, are instrumental for the advent of the above emerging technologies.

## 2. New highly efficient materials

**Figure 3.**
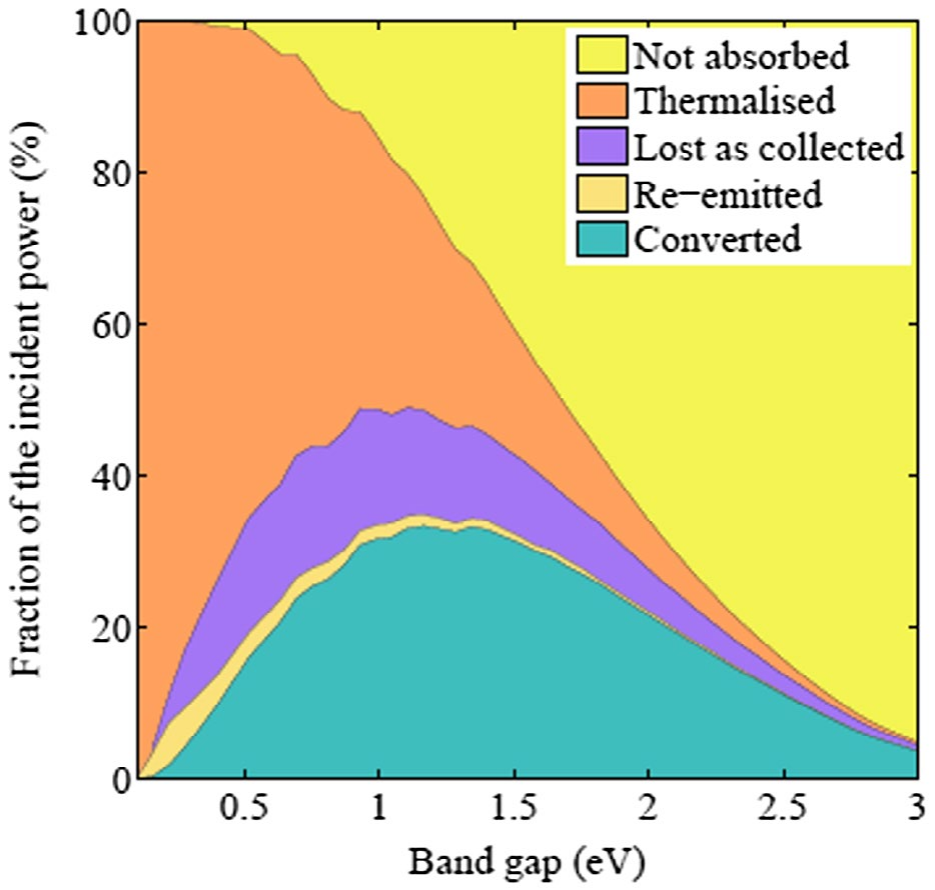
The incident power is only partially converted even in ideal devices. The plot shows how devices in the Shockley–Queisser limit convert sunlight (AM 1.5 standard spectrum) and how the remainder is split between different losses as a function of the fundamental absorption band gap of the device: (i) the light may not be absorbed (yellow), (ii) the excess energy of the absorbed photon is turned into heat (orange), (iii) recombination of electron and holes results in an emitted photon (green), (iv) the flow of charges to the electrodes generates entropy (blue).

The main energy losses during the conversion of solar energy to electrical power by a solar cell are transmission losses and thermalization losses, with different relative contributions as a function of the semiconductor bandgap (see Figure 3 below). Several strategies exist to reduce those losses, such as multijunction cells, hot-carrier solar cells, intermediate band solar cells, or multiple exciton generation.

### 2.1. Multijunctions

As of today, multijunction cells are the only concept which demonstrated performances overcoming the single-junction Shockley–Queisser limit and reached industrial applications.

**Figure 4.**
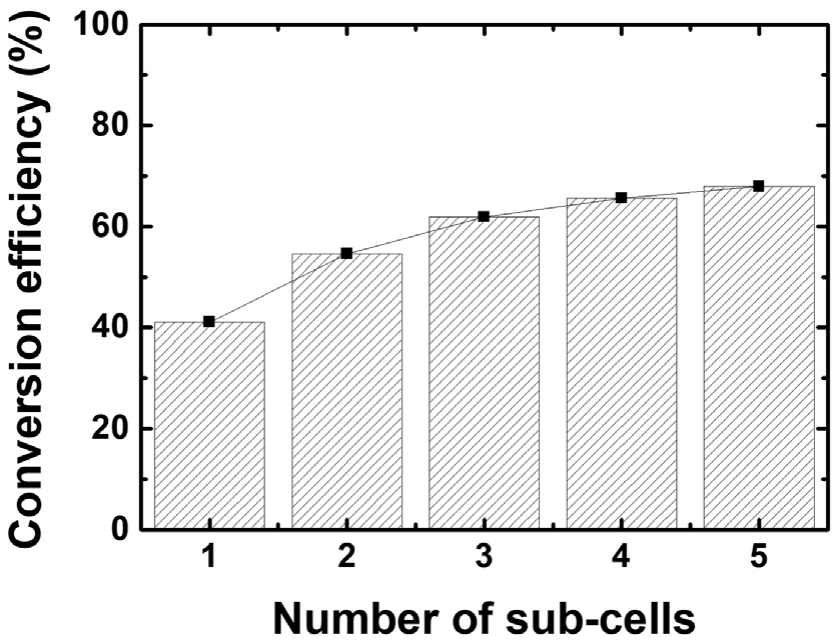
Output power provided by multijunction cells in the radiative limit, as a function of the number of subcells, under AM1.5d for a 1000 sun concentration. Data from [22].

The idea of multijunction devices is based on the fact that a cell reaches its maximum conversion efficiency for photons at a wavelength that is equal to its bandgap, when thermalization and non-absorption are inexistent. Dividing the solar spectrum into several wavelength ranges, and converting those wavelength ranges with distinct cells of suited bandgap, allows to reach higher efficiencies, as shown in Figure 4. Splitting of the incident spectrum could be achieved with bandpass filters (not reviewed here, see e.g. [19–21]), or by stacking different materials, the highest bandgap material being on top of the device.

Stacked junctions can be considered in two configurations, so-called 2-wire and 4-wire configurations (also commonly referred to as 2- and 4-terminals). In the 2-wire configuration, the different cells of the stack are connected in series. As a consequence, the device reaches its optimal performance when the current in the different subcells are equal. Otherwise, the cell that delivers the lowest current will limit the current delivered by the complete device, and the excess current produced by the other cells is wasted. In order to achieve a series connection between the different junctions of the stack, highly recombinative layers are used, such as tunnel junctions, which should allow large currents with moderate voltage drops. In the 4-wire configuration, the subcells are separately connected. This relaxes the necessity of current matching, but other difficulties emerge. Because current is separately extracted from each cell, low resistivity layers and contact grid are required in between to allow lateral currents. Those layers need in addition to be highly transparent. In both the 2- and 4-wire configurations, complexity also arises from the compatibility of the different materials that are included in the device, in terms of intrinsic properties (such as the lattice constant for monolithic III–V cells) or processing steps.

Different families of material have been used for multijunction cell fabrication. III–V materials (compounds of elements of columns III and V in the periodic table) have been widely considered, since they provide a large spectrum of available bandgaps covering the sun spectrum. Moreover, efficient fabrication methods are available, and many compounds provide direct bandgaps (which are preferred, since their high absorption allows fabricating thinner cells, requiring less material and relaxing the constraint on transport properties). Nevertheless, one limitation is the requirement of lattice matching for keeping high material qualities. A material combination that fulfills this condition is the Germanium/GaAs/InGaP cell. Although it allows reaching high performances (41.6% under 364 suns have been reported [23]), this cell is far from the condition of current matching. The bandgap of the germanium cell being rather low, it produces a current almost twice as large as the limiting subcell current. A device with the optimum combination of materials is therefore not readily available, but can be approached thanks to various technological strategies: wafer bonding [24–26], metamorphic growth [27], inverted stacks [28–30], dilute nitride [31], and multiquantum wells [32–34].

Few of these strategies have been successful, and led to the highest reported conversion efficiency to date, at 46% under 508 suns ([26], using wafer bonding). Nevertheless, the high cost of those devices prevents their application as flat panels [35,36]. They can find commercial applications for concentrated photovoltaic (CPV), where the surface of the cell is much reduced, or for space applications where the meaningful indicator is the power produced for a given weight. Because the crystalline substrate is an important part of the final cost, it has been proposed to substitute the Ge substrate and cell by silicon.

The band gap of silicon cells can cover relevant bandgap values by combining different phases from crystalline to completely amorphous [37–39]. Compared to III–V materials, silicon is abundant, and low-cost processing is available, but reaches much lower efficiencies (13.6% [38]). Those cells can be made flexible, with various colors and shapes, so that they can find application in specific cases such as building integrated PV (BIPV). Nevertheless, research on micro-crystalline and amorphous silicon devices has attracted less interested in the past year due to the rise of attractive alternatives such as perovskite cells.

Around 90% of the market is constituted by silicon cells [40]. In laboratories, the 25% efficiency threshold has been reached in 1999 [41,42], and slight improvements have been observed since, up to more than 26.6% [43]. Those values approach the theoretical efficiency of the silicon cells (29.4% [44]). The silicon technology is therefore reaching a plateau, pointing toward the need of new conversion concepts such as multijunctions, considering that the bandgap of silicon is not far from the ideal for the bottom cell of a dual junction device. Experimentally, only integration of III–V on silicon has reached efficiencies higher than the record silicon cell alone [25,45]. Theoretically, current perovskites solar cells should also lead to efficiency improvements [46]. Although this has not been reported so far, encouraging results have been demonstrated (with a current record at 23.6% [47]). Thin film multicrystalline large gap CIGS alloys present rather low efficiencies, so that their integration on silicon is not currently an option [46,48].

### 2.2. Ultrathin cells

In conventional cells, the limitation factor for the cell thickness is related to its absorptivity: the cell needs to be thick enough so that, over the desired bandwidth, most photons will be absorbed and generate an exciton. For direct bandgap III–V semiconductors, this translates into a thickness of a few microns. Our objective is to gain 1–2 orders of magnitude in thickness without detrimental effect on the solar cell absorption, which means an absorber thickness below 100 nm for GaAs solar cells. The interests that drive the research toward ultrathin solar cells are numerous and can be grouped along two main axes.

First, a thinner cell can contribute to higher efficiency. Same absorption over a smaller thickness means higher optical intensity, which has a positive influence over the open-circuit voltage (*V*_oc_) of the cell. Concentration moreover enhances the efficiency of nonlinear processes like the two-photon absorption in intermediate band solar cells (IBSCs). Having thinner cells also means the carriers are generated closer to the contacts, limiting the volume recombination. In addition, this is a mandatory requirement for hot-carrier solar cells (HCSCs), for which the extraction time has to be reduced drastically in order to prevent the thermalization of the conduction electrons.

Secondly, materials used for III–V solar cells can be quite expensive, and a reduction of the thickness has a direct impact over the global cost of the cell. In addition, those cells being usually grown epitaxially at a growth rate about 0.1 nm/s for molecular beam epitaxy, the growth duration can be considerably reduced in the case of ultrathin cells, making them furtherly economically viable. Also, the complex material arrangement often required for high-efficiency concepts makes the cells difficult to grow with a high crystallinity over more than 100 nm.

#### 2.2.1. Epitaxial lift-off

High-quality crystalline thin films are grown on thick substrates (a few hundred micrometers) which account for a significant part of the total cost of the cell. In order for the absorber thickness reduction to have an economic impact over material usage, it is thus essential that the substrate could be used for multiple growth runs [49].

This is the purpose of epitaxial lift-off (ELO), by which a release layer is grown in-between the substrate and the device. By the combination of a chemical reaction and a strain, the release layer is etched, resulting in a device separated from the substrate. The difficulty lies in the ability to produce no-cracks and low roughness layers in a reasonable amount of time.

The main path relies on the use of an AlAs sacrificial layer in combination with a high-selectivity HF etchant for the release of layers grown on a GaAs substrate. In order to be able to under-etch over the whole wafer, a mechanism is required to maintain a path open for the etching solution to reach the release layer. A possible method is called weight-assisted ELO, whereby the device layer is fixed to a support, and a weight is added to the substrate layer [50].

Other approaches are currently considered, like the replacement of the release layer/etchant couple with InAlP/HCl. It leads to potentially cleaner surfaces after etching, but necessitates rethinking the materials used in the device. Also, other strains can be applied to separate the device layer, for example, surface tension ELO [51].

#### 2.2.2. Light trapping

As seen earlier, ultrathin absorbers imply light concentration. A mirror at the back of the cell doubles the effective distance traveled by light. With a random surface texturing, the limit for light path enhancement is *F* = 4*n* ^2^, where *n* is the real part of the refractive index of the material [52]. Multiresonant absorption is another way for light trapping in direct bandgap semiconductors with sub-wavelength thickness [53]. All those light-path enhancement strategies require the presence of a mirror at the back of the cell, which is yet another motivation for developing ELO.

Multiresonant absorption requires periodical or pseudo-periodical nanopatterns with dimensions close to the wavelength. The grid can be implemented in a number of ways, at the top or the bottom of the cell, and can be made of metallic or dielectric material [54]. A classical approach is to use a metallic pattern at the back side of the cell, as we need the back surface to be a mirror anyway [55,56].

This back mirror is deposited before the ELO process, for example, using soft nanoimprint lithography. First, a thin (about 100 nm) layer of dielectric material (TiO_2_ sol–gel) is spin-coated over the device, and a soft PDMS mold, replicated from a silicon master, is applied onto it. The solvent containing the TiO_2_ is evaporated through the mold, and the remaining TiO_2_ is solidified by application of a heating treatment. Then, the mold is removed, leaving nanopatterns on the surface. The whole substrate is then covered by a 200-nm layer of metal (gold or silver). Finally, by applying the ELO process presented earlier, we release the device layer and obtain a cell with a nano-structured back mirror.

Light management is especially interesting for solar cells with quantum structures like multiple quantum wells (MQW), superlattices [57] or multi-stacked quantum dots [58]. Indeed, a smaller number of quantum layers is favorable for an improved carrier transport and for the reduction of dislocation density. We apply this approach to several potential applications, especially for the spectral region covered by quantum dots (QDs) where absorption is notoriously weak (less than 1% per quantum confined layer).

Fabrication of MQW solar cells has been reported [59]. Those MQW are comprised In_0.18_Ga_0.82_As wells surrounded by GaAs_0.78_P_0.22_ barriers, and were inserted in the i-region of a GaAs–p-i-n junction. A special care was taken to balance the strain induced by wells that have some lattice mismatch with GaAs.

**Figure 5.**
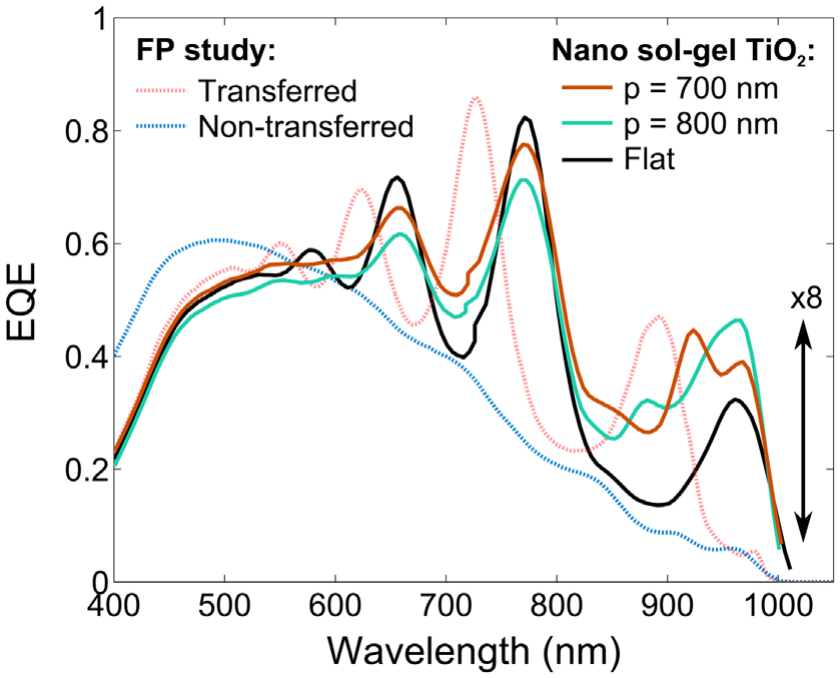
EQE measurement of MQW for non-transferred and transferred solar cells, with different types of back mirrors. FP stands for Fabry–Perot resonance.

In Figure 5, the absorption of those structures is compared before and after transfer, and for different nano-structured back mirrors. The difference between ‘Flat’ and ‘Transferred’ is the presence of a 100-nm layer of TiO_2_ at the back of the former; p indicates the period of the nanostructures.

Compared to the non-transferred solar cell, a maximum of ×8 external quantum efficiency (EQE) ratio enhancement is obtained for a wavelength of 965 nm, while the flat EQE indicates a maximum of ×5.6 ratio enhancement for the same wavelength position. Therefore, the addition of the nanogrid at the back results in a maximum of ×1.5 ratio enhancement. These results are coherent with the electromagnetic calculation made using rigorous coupled wave analysis (RCWA). This structure still requires numerous improvements to hopefully reach the full potential of multiple resonance, such as deposition of an anti-reflection coating (ARC), and optimization of the nanogrid parameters and deposition method.

**Figure 6.**
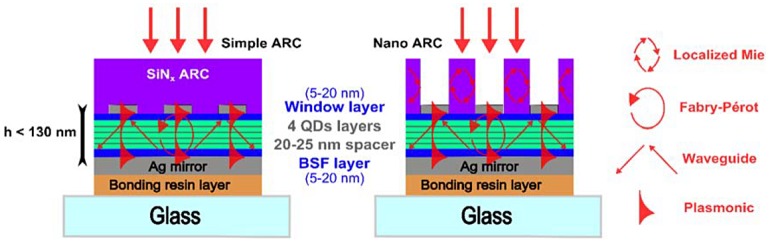
Examples of designs for ultrathin QDSCs benefitting from different resonance mechanisms in order to obtain high broadband absorption.

Several options are considered in order to develop ultrathin heterostructures. For QDSCs based on the concept of intermediate absorption, the absorption must be enhanced in three spectral domains covering the transitions between valence and conduction, valence and intermediate, and intermediate and conduction bands. Taking advantage of different types of resonance mechanisms could be the way to go to reach high absorption rates over all those spectral domains (see Figure 6). Calculations have already provided convincing results, supporting that approach.

#### 2.2.3. Conclusion

Achieving ultrathin solar cells is a goal relevant to the whole field of III–V cells provided they can be made cost effective and very absorbing. Ultrathin technology will lead to better material usage, better carrier collection, and higher open-circuit voltage, ultimately increasing the efficiency and reducing the cost of the cells. Finally, it is a necessary brick for the development of IBSC and HCSC.

### 2.3. Hot-carrier solar cells – concept

Hot-carrier solar cells are a remarkably elegant concept to achieve a solar energy conversion close to the Carnot efficiency [60,61]. A simple yet challenging idea to avoid thermalization losses while keeping a narrow bandgap to increase absorption (see figure losses) would be to selectively collect carriers before their relaxation with the lattice vibration, i.e. as they while they are still ‘hot’. Such HCSC concept was first described by Ross and Nozik [60] ignoring Auger mechanisms, and Würfel later revisited the concept considering impact ionization as a dominant process [61].

Two main requirements are needed to achieve power generation from the extraction of hot carriers: a slow carrier cooling absorber combined with energy-selective contacts (ESC). Relaxation of the photocarrier population toward an equilibrium state, through elastic carrier–carrier interaction, should be the dominant process so that a hot-carrier population is obtained under continuous illumination. While this is the case in most photovoltaic materials, and especially for epitaxially grown III–V materials as those on which we will focus in the following discussion, this condition may not always be fulfilled, especially in the case of quantum structures where Auger phenomena could be the dominant process [62]. Such aspect being of marginal concern in the research on HCSC, it will not be discussed furthermore in this paper. Our approach at NextPV lab is to develop and optimize in parallel hot-carrier absorbers and ESC to understand and validate the operation of each element, before combining both in a future complete proof-of-concept device.

#### 2.3.1. Hot-carrier solar cells – absorber

**Figure 7.**
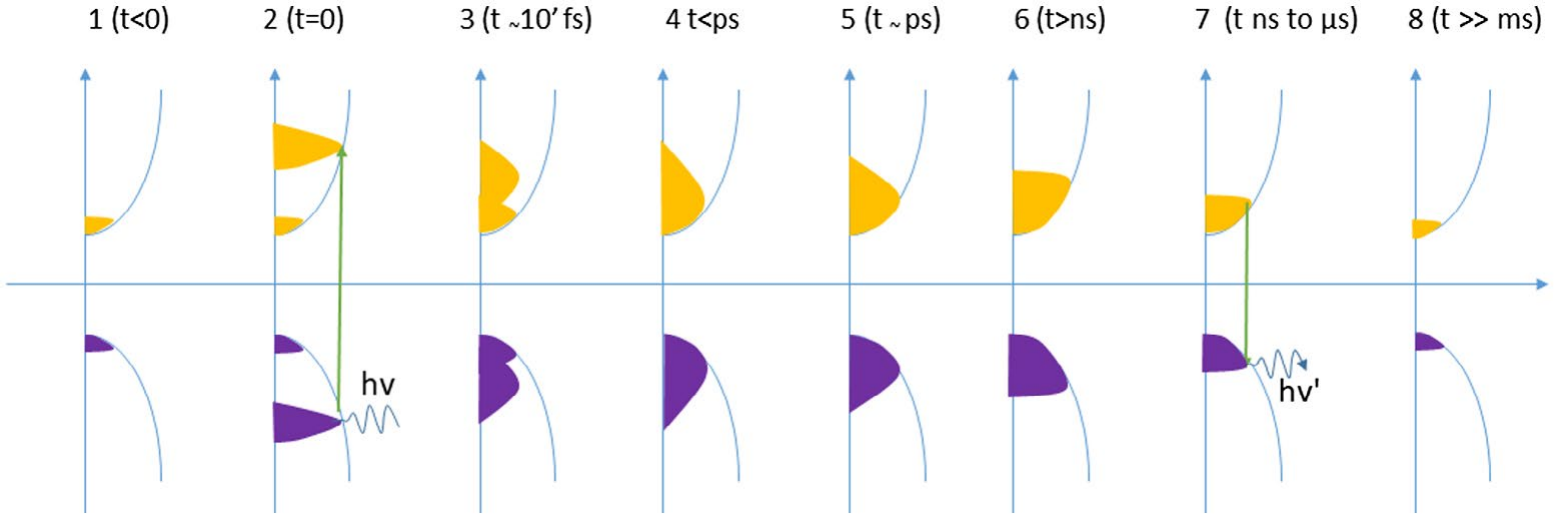
Time evolution of the electron and hole population (vertical axis: energy, horizontal axis: carrier concentration) after laser excitation, at different time steps adapted from [79].

Slow carrier cooling absorbers are necessary to obtain a stable population at a temperature higher than that of the lattice. It is now well understood that photocarriers lose their energy excess through an interaction with longitudinal optical (LO) phonons, as demonstrated in GaAs by Shah and Leite [63]. Figure 7 shows the time evolution of a carrier population from a laser excitation. Before illumination (1), a small number of electrons exist in the conduction band, and are in thermal equilibrium with the lattice. Just after absorption, the photocarrier distribution is a non-equilibrium one and corresponds to that of the absorbed radiation: a temperature cannot be determined at this stage (2). The distribution then reaches an equilibrium state (3), (4) through elastic carrier–carrier scattering, with a characteristic time in the order of less than a picosecond [64]. The carrier’s distribution at this stage has a higher temperature than the lattice, and it is the step at which HCSC should operate to overcome the Shockley–Queisser limit. Interaction with LO phonons then cools down the distribution toward the lattice’s temperature (steps (5), (6)) before the onset of radiative recombination shown steps (6), (7) (t≥1ns), finally leading to state (8), similar to configuration (1). One of the challenges of HCSC is therefore limiting the thermalization rate of the hot carriers’ distribution (3), (4), and phonon engineering is a straightforward idea to do so. The question of the decay of LO phonons into longitudinal acoustic (LA) phonons through the Klemens mechanism [65] is critical as it provides a pathway to achieve a so-called bottleneck effect, leading to a hot-phonons population in thermal equilibrium with the hot photocarriers [66].

Using III–V materials, quantum structures were shown to significantly decrease the cooling rate of photocarriers [67,68]; particularly, Rosenwaks et al. [69] observed a significantly broader emission distribution in the time-resolved photoluminescence (TR-PL) spectra of GaAs/AlGaAs multiquantum wells for high carrier injection (above 10^18^ cm^−3^), and ascribed the phenomenon to a hot-phonon bottleneck effect. Hot-carrier relaxation dynamics in quantum systems was later summarized by Nozik [70], also highlighting the importance of Auger mechanisms as they could potentially break the phonon bottleneck.

In the previous decade, the bulk of the work on hot-carrier absorber has been focusing on ways to quench Klemens mechanism through a so-called phononic bandgap (between the highest LA and lowest LO phonon energy), which can occur in some crystals with a large mass difference between the cation and the anion [71]. Quantum structures based on III–V materials, particularly multiquantum wells, have remained the most studied pathway to obtain slow carrier cooling absorbers. Le Bris et al. investigated on GaSb-based heterostructures under continuous wave illumination [72], from which the influence of the nanostructuration of the absorber on the carriers’ temperature was studied. It was also noted that confinement of photocarriers in a limited volume with the use of large bandgap barriers (‘claddings’) helps to limit the heat flow at each extremities of the absorber, thus significantly enhancing the temperature of the photocarriers’ distribution.

More recently, alternative materials have been considered as hot-carrier absorbers; metals [73], in the absence of an optical bandgaps, can potentially absorb the full solar spectrum and transfer the resulting high-energy photoelectrons (only relevant carriers in this specific case) to a semiconductor with an electronic affinity smaller than the work function of the absorbing metal. More recently, very interesting results have been obtained in perovskite materials with the observation of a hot-phonon bottleneck [74] and thermalization properties comparing even favorably to bulk GaAs. Also, colloidal perovskite nanocrystals were recently found to be of major interest in the scope of HCSC realization with a tentative full device presented and characterized by Grätzel’s group [75].

A straightforward path to determine the carriers’ temperature derives from the generalized Planck law, which accounts for the photoluminescence signal [76]. Under sufficient pump power, a broadening of the high-energy tail of the PL spectrum is a marker of a hot-carrier population [66].

(1) $$I_{\text{PL}}\left(\hbar \omega \right)=A\left(\hbar \omega \right){\left(\hbar \omega \right)}^{2}{\left(\text{exp}\left(\frac{\hbar \omega -\Delta \mu_{e-h}}{kT_{e-h}}\right)-1\right)}^{-1}$$

The temperature can be determined with a rough precision of ∼∓20K from the linear fitting of the high-energy tail of the PL signal, with a first-order development of equation (1), where *I*_PL_ is the PL intensity, *A* is the absorptivity, Δ*μ*_*e*−*h*_ is the difference in chemical potential between electrons and holes, and *T*_*e*−*h*_ is the temperature of the radiation (mostly ascribed to an electronic temperature). A thermalization factor *Q* (in W.K^−1^/cm^2^) is introduced to characterize and was later used as a benchmark characterizing the capacity of a material to slow the cooling of a high-energy photocarriers’ population [77,78]. An absorber is generally considered to be slow cooling for a Q value below 100 W.K^−1^.cm^−2^ [79]. In most cases, one assumes that a significantly larger part of the thermal energy is distributed to the electron rather than the holes, owing to their difference in effective masses. This approximation has been challenged by Gibelli et al. [80] by introducing a two temperatures generalized Planck law, as well as the necessity of a full-spectrum fitting for a more quantitative determination of the carrier cooling properties of a material [81].

**Figure 8.**
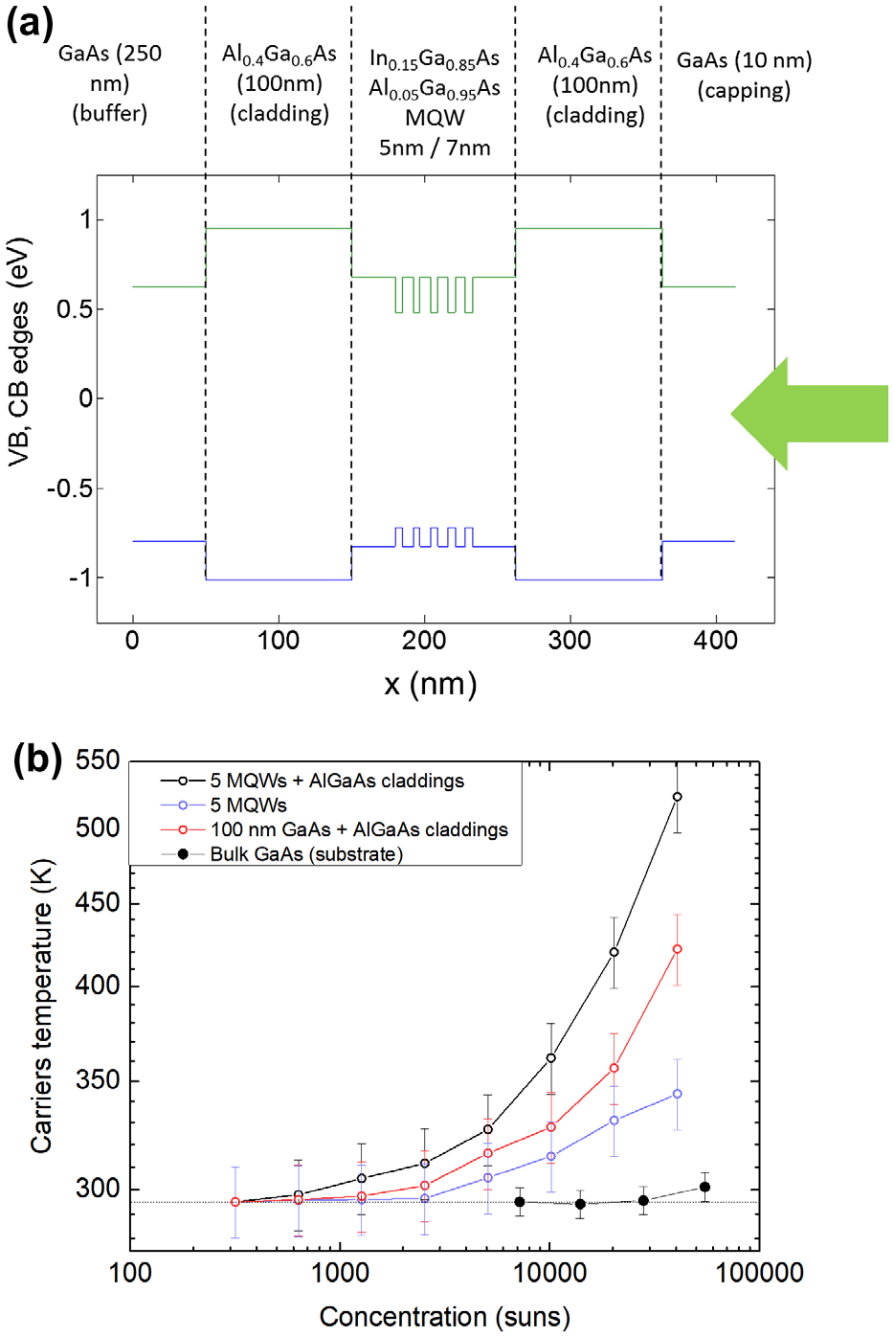
(a) Slow carrier cooling absorber based on GaAs/InGaAs multiquantum wells structure including AlGaAs carrier confinement claddings. (b) Photocarriers’ temperatures determined from the PL analysis of different samples for illuminations varying from 100 suns up to 50,000 suns.

While the realization of hot-carrier absorbers and the understanding of cooling mechanisms involved in bulk and nano-structured materials remain of major interest for the field, it is already possible to obtain materials that could readily be used in a complete proof-of-concept device with the aforementioned slow cooling properties. We oriented our researches toward nanostructured III–V materials, deposited by molecular beam epitaxy (MBE), owing to their demonstrated excellent and easily tunable carrier cooling properties, and their straightforward integration in a tentative complete HCSC device [82]. In Figure 8(a) the band diagram of a typical hot-carrier absorber test structure is shown. The material stack consists in a five-MQW region of In_0.15_Ga_0.85_As/Al_0.05_Ga_0.95_As (5 nm/7 nm, respectively) with a total thickness of 100 nm (spacing Al_0.05_Ga_0.95_As layers are including at each extremities) between two 100-nm Al_0.4_Ga_0.6_As claddings. The continuous illumination comes from a 532-nm laser in a classical confocal setup at room temperature, and the photoluminescence signal is analyzed using the generalized Planck law from which the carriers’ temperature *T*_*H*_ and the thermalization coefficient *Q* are extracted. The illumination is varied from 100 suns to 40,000 suns. Figure 8(b) shows the evolution of the photocarriers’ temperature as a function of the concentration comparing different heterostructures: MQWs with claddings, MQWs without cladding, bulk GaAs with claddings and bulk GaAs. Consistently to what was hinted by Le Bris [72], the combination of MQWs and large barriers embedding the absorber leads to higher temperatures (520 K for 40,000 suns); the role of the claddings is avoiding carrier scattering outside of the absorber limits. Hot carriers are also observed in MQWs without cladding albeit at a markedly lower temperature (400 K). It should also be noted that a roughly similar observation can be made for bulk GaAs embedded between two claddings, showing that hot carriers can be generated in a relatively simple design. While not the highest performing solution, this provides a pathway simplifying the realization of a proof-of-concept device. The bulk GaAs layer is shown for comparison, and we see that in the absence of nanostructuration or carrier confinement from claddings, the temperature of the photocarriers remains that of the lattice (room temperature). The dominant interpretation of the effect of the slowed photocarrier cooling in MQWs absorber is that quantum confinement may inhibit the thermalization pathway [71,83] through a phonon bottleneck, though recent theoretical work has been challenging this interpretation, instead asserting that a change in the carrier-to-phonon LO interaction is a more likely process [84].

As the absorbers that were investigated in our laboratory were based on nanostructured III–V materials, it makes sense to develop energy-selective contacts epitaxially grown on similar substrates and using the same deposition method, thus allowing a one-step realization of a complete structure.

#### 2.3.2. Hot-carrier solar cells – Selective Energy Contacts

As compared to the absorber part, the research on selective energy contacts is somewhat scarcer albeit different concepts having been considered. Ideally, the hot photocarrier would transit from the absorber to the collector (typically having a wider bandgap than the absorber) through a single energy state, and transforming the excess in kinetic energy (heat) into potential energy (voltage); an energy-selective contact can be viewed as a thermoelectric leg for which it is possible to calculate a Seebeck coefficient [85].

Theoretical evaluations of the required properties of an SEC, as well as the influence of the contact’s parameters on the performance of a final device have been extensively studied by Le Bris [79,86], as well as O’Dwyer et al. [87], mainly insisting on the importance of carrier selectivity (energy width of the contact) and extraction level in regard to the photocarrier’s temperature in the absorber. Also, Takeda [88] used the example of resonant tunneling diodes (RTDs) in the detailed balance model to discuss on the requisite of the SEC conductance, concluding that it must be within a suitable range, and the expected efficiency of an HCSC would be negatively impacted for a too high or too low conductivity. While a too low conductance can easily be understood in terms of series resistance effect, a too high conductance is also detrimental for the efficiency as the temperature of the photocarriers’ population will decrease as compared to the optimum extraction energy.

Various ideas have been considered to achieve the feature of energy selectivity. A straightforward approach is to use low-dimensional structures such as quantum dots [89,90] for a 3D confinement, or double-resonant tunneling barriers (DRTBs) [82,88,91] for a 1D confinement. Both structures lead to the apparition of a confined state through which photocarriers can selectively be extracted. Experimentally, the well-known negative differential resistance (NDR) effect is observed, the peak current then corresponding to the resonance current at the first order. Other original pathways to energy selectivity have also been considered, such as ‘optical selectivity’ [92] which consists in a narrow bandgap hot-carrier absorbing material with a broadband absorption profile and a narrow band emission profile; the resulting emitted light is then collected in an integrated PV solar cells with a larger bandgap, matched with the maximum of the narrow band emission. Although seemingly complicated to experimentally realize, such concept shows the potential variety of solutions that could be considered for converting the excess of photocarrier’s heat into potential energy.

DRTBs are an attractive solution in the scope of realizing a proof-of-concept HCSC, owing to their apparent simplicity, the extensive knowledge on such heterostructure and their compatibility with a MBE process based on III–V materials. Most approaches consider that the amplitude of the transmission remains unchanged by the application of an external voltage on the heterostructure [93]. We were particularly interested in the symmetry breaking of the DRTB resulting from such bias; indeed, as the maximum of the transmission is related to the ratio of the transmission of each individual barrier [94], a substantial decrease of the DRTB performances is expected which may limit the resonant current particularly at room temperature where HCSC are supposed to operate. This issue has previously been addressed by Allen [95], proposing a concept of ‘effective barrier symmetry’ by offsetting the breaking through asymmetric structures.

**Figure 9.**
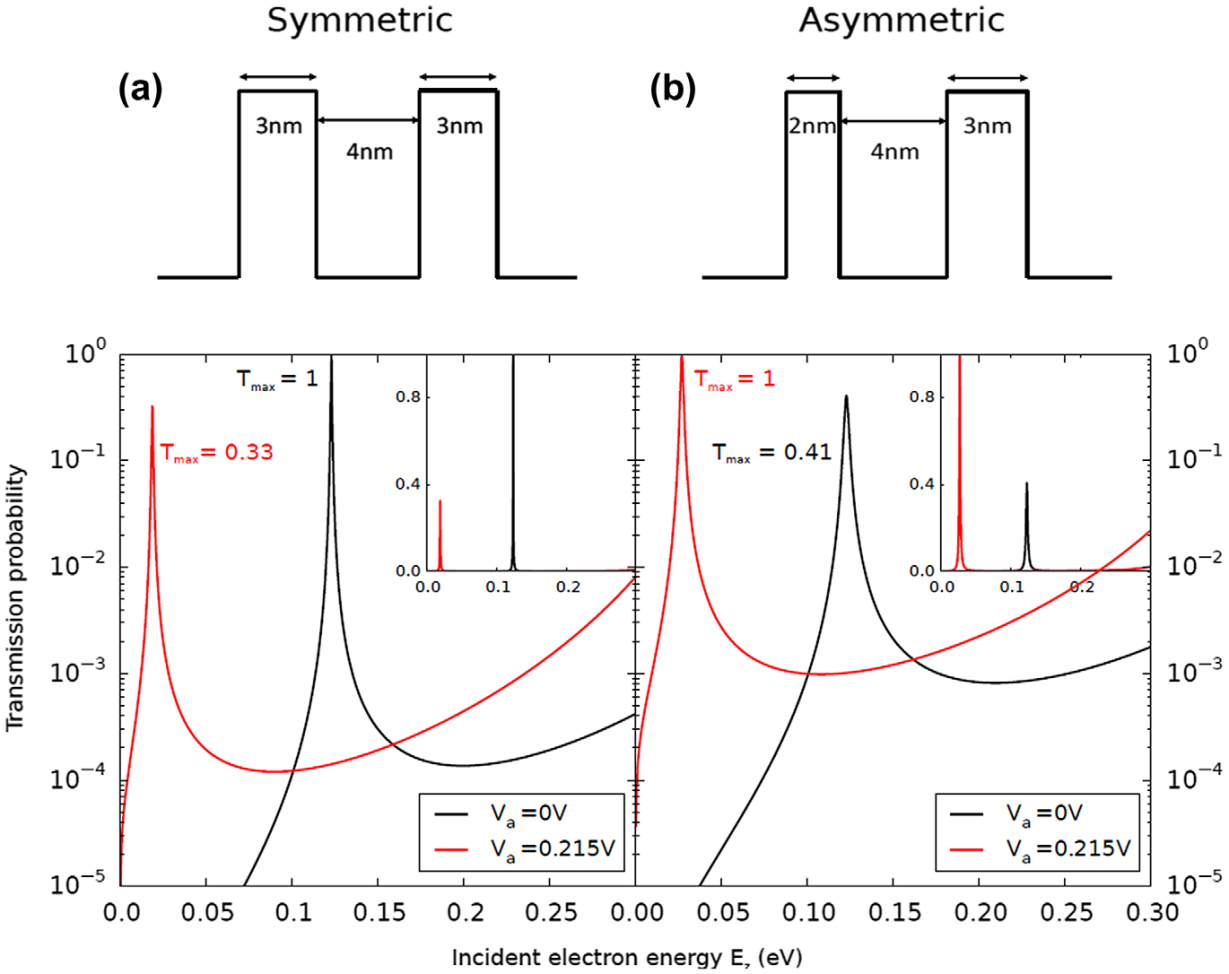
Modeled electronic transmissions of symmetric (a) and asymmetric (b) Al_0.6_Ga_0.4_As/GaAs/Al_0.6_Ga_0.4_As structures when no bias is applied (black curves) and under a 0.2 V bias (red curves). Inset: similar curves in linear scale (from [96]).

A numerical modeling of the electronic transmission of symmetric and asymmetric Al_0.6_Ga_0.4_As/GaAs/Al_0.6_Ga_0.4_As structures [96] is shown Figure 9. As expected, the maximum of the transmission is equal to unity for the symmetric structure, but the polarization of the structure leads to a significant decrease. For the asymmetric structure, while the transmission’s maximum is much lower than 1 when unbiased, the application of an external voltage leads to a full recovery of the transmission’s maximum close to 1. This highlight an often-overlooked characteristic of DRTB which is of major importance for an application to HCSC: the tunnel resonance (energy at which the photocarrier’s energy matches that of the confined state) and the feed resonance (voltage at which the resonant population difference between the absorber and collector is maximal) are not the only resonances that need to be taken in account, and amplitude resonance (the voltage at which the amplitude of the transmission is maximum) is a critical parameter for optimizing the carrier transport through a DRTB. We also showed that the simultaneous optimization of these three resonances allows for a higher energy selectivity level through the contact which is of major importance to maximize the potential efficiency increase that HCSC may bring as compared to conventional solar cells [97].

**Figure 10.**
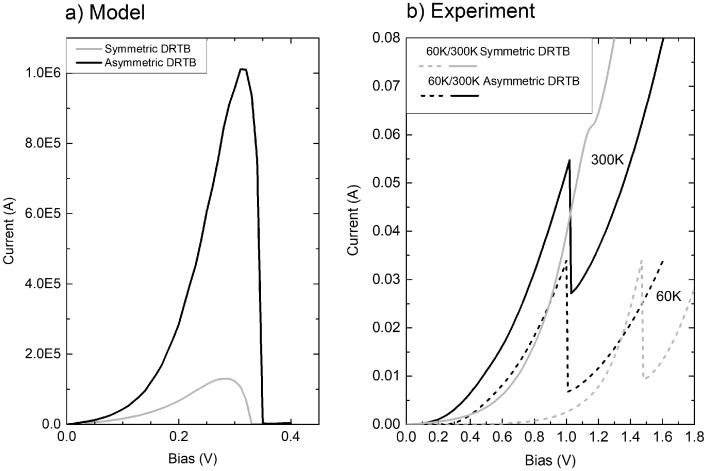
(a) Modeled tunnel current of a symmetric (grey curve) (resp. asymmetric, black curve) 6 nm/3 nm/6 nm (resp. 5 nm/3 nm/6 nm) Al_0.6_Ga_0.4_As/GaAs/Al_0.6_Ga_0.4_As double-resonant tunneling barrier; (b) Experimental current–voltage characteristics of similar heterostructures measured at 60 K (dashed curves) and 300 K (solid curves).

Experimental samples have been realized by MBE and analyzed by temperature-dependent current–voltage analysis (J–V–T). Figure 10(a) presents a comparison of modeled J–V curves for symmetric and asymmetric structures, showing an expected higher resonant current for the latter. Experimental curves are shown in Figure 10(b) for 60 K and 300 K. While both samples have a roughly similar behavior at low temperature, the NDR is fully preserved at room temperature for the asymmetric structure while completely vanishing for the symmetric one, in excellent qualitative agreement with the conclusions from the model. This further confirms the beneficial effect of using asymmetric structure for a better matching of the transmission’s amplitude resonance with the tunnel and feed resonances [97], and allows a room temperature operation of the SEC. In the future, selective contact modeling should be conducted within the non-equilibrium Green’s functions framework which allows to add the electron–phonon scattering in order to be more realistic.

#### 2.3.3. Hot-carrier solar cells – conclusion and perspectives

It has been illustrated that the two critical parts of a HCSC are already functional under the normal solar cell operation conditions (continuous illumination and room temperature). Carriers’ temperatures significantly higher than that of the lattice have been experimentally measured, and electrons’ transport through a single energy state has been observed, both features on sample stemming from a similar experimental MBE process on GaAs substrate. The next logical step is the combination of both parts in a single-step device where hot carriers (electrons) would be optically generated in a hot-carrier absorber, then extracted through a double-resonant tunneling barrier with an optimized geometry to maximize the resonant current at room temperature. The extraction of energetic particles from a hot-carrier population also brings several questions regarding the influence it will have regarding the said population, and especially potential changes of its equilibrium temperature. Those questions, along with an experimental realization of a complete proof-of-concept device, will hopefully be addressed during the upcoming year.

## 3. New materials for solar competitiveness and improved integration

### 3.1. Dye-sensitized solar cells

Dye-sensitized solar cells (DSCs) [98], was regarded as one of the most promising photovoltaic devices, have been extensively investigated due to their high theoretical efficiency, facile fabrication processes, and potential low cost. These solar cells use molecular dyes to absorb the solar spectrum efficiently. The dye molecules are bound to TiO_2_ (mesoporous) and the porous network is then infiltrated by either an electrolyte or a conducting polymer. The exciton generated in the dye is then dissociated as the electron is very quickly (tens of fs) injected in the oxide. The dye cation is regenerated by either a redox shuttle in the electrolyte or a hole conducting polymer.

Over the past two decades, continuous research efforts have contributed to the significant advances in the DSC performance [99]. To date, DSCs devices employing a zinc–porphyrin-based co-sensitized system have shown an efficiency record of 13% using liquid electrolytes, which may limit their outdoor applications [100]. Practical advantages have been gained by replacing the liquid electrolyte with an organic hole transporting material (HTM) [101–103] such as spiro-OMeTAD (2,2′,7,7′-tetrakis-(N,N-di-p-methoxyphenylamine)-9,9′-spirobifluorene), which is the most widely used. The results reached with this kind of solid HTM have caught up those with liquid cells, with power conversion efficiencies over 10% [104].

The concept of hybrid organic–inorganic solar cells is based on the sensitization of porous semiconducting metal oxide films with organic or metallo-organic dyes. DSC’s technology benefits from fabrication through abundant, nontoxic, and cheap materials, as well as tunable color depending upon the dye employed and relatively high-power conversion efficiencies (PCEs up to 14.0% [105] for liquid and 7.5% [106] for solid state devices at the laboratory scale). In particular, advances over past years have been achieved by optimization of the redox mediator nature along with the development of new ruthenium-free dyes leading to record PCEs over 13% for small area devices, making them competitive with amorphous silicon-based devices [100,107]. To push further the power conversion efficiencies, co-sensitization with complementary dyes of semiconducting oxide porous layers combined with a fine tuning of the electronic properties of each dye appeared to be a very promising route [105,107,108]. Many orange and red chromophores have been therefore designed for DSCs, whereas examples of efficient green or blue dyes are rarer [109] and usually involved long and costly preparation and purification routes.

**Figure 11.**
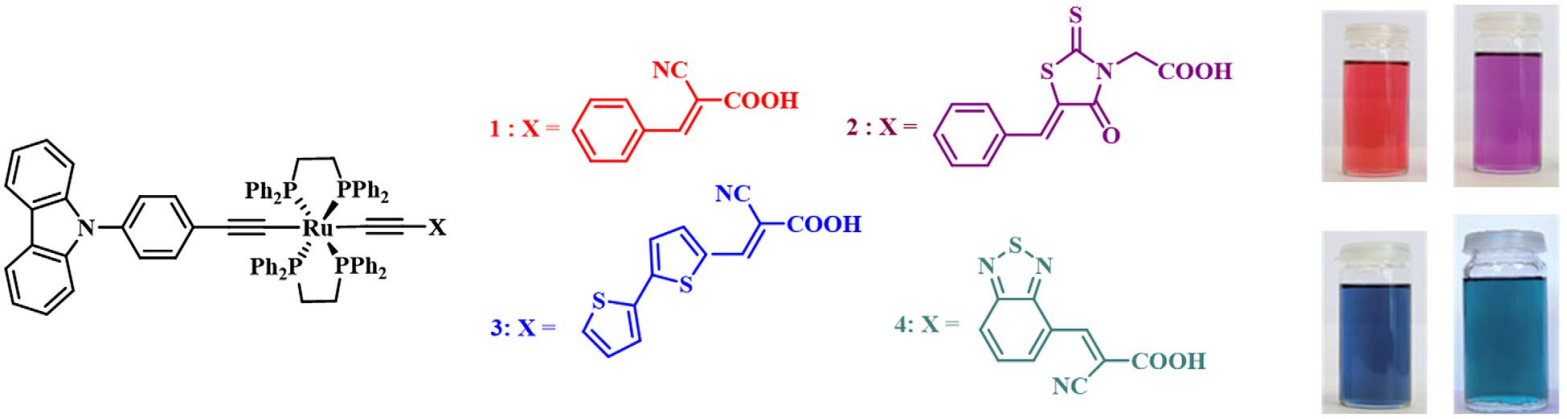
Schematic representation of the molecular formula of Ru–diacetylide complexes 1–4.

In this context, the ISM team has developed an original D-π-[M]-π-A architecture including a ruthenium–diacetylide, i.e. [M] = [Ru(dppe)_2_] where dppe is bisdiphenylphosphinoethane, embedded within a push–pull structure which were prepared using convergent synthetic routes [109,110]. Fine control of the electron-withdrawing properties of the π-A units led to a new family of chromophores showing red, violet, blue, or greenish color and the optoelectronic properties of which are suitable for using in DSCs (Figure 11). Thus, these dyes led to PCEs above 6% in standard single-dye DSC and up to 7.5% in devices involving photoanodes co-sensitized with dyes **1** and **3**.

**Figure 12.**
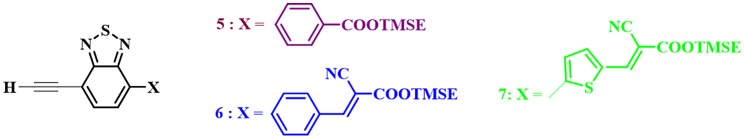
Schematic representation of the molecular formula of new acceptor building blocks 5–7 (protected form with TMSE = 2-(trimethylsilyl)ethyl).

Presently, new organometallic dyes built from the D-π-[M]-π-A structure including various acceptor moieties based on the 2,1,3-benzothiadizole unit are under investigation. First of all, three new acceptor building blocks have been synthesized (Figure 12).

After coupling with a ruthenium–vinylidene complex endowed with a N-(phenyl)-carbazole group and deprotection of the carboxylic acid function, both green and blue dyes were obtained, the electronic properties of which were fully characterized by UV–visible spectroscopy, cyclic voltammetry and density functional theory calculations. Preliminary characterization in DSCs revealed promising performances for green or blue dyes [111].

As liquid electrolyte, yielding to more efficient devices, were found to induce difficult issues for commercialization. The NextPV laboratory has focused its work on solid-state solar cells and on process optimization for the preparation of DSCs and perovskite solar cells.

In a first step, simple and efficient dyes based on indoline donor units having fluorene substituent group and acceptor units have been applied as light harvester materials in DSCs [112]. For further improvement of the DSC performance, additional substituent groups with long alkylene chains were introduced to the fluorene substituent group or to acceptor units in order to extend the absorption spectrum and to suppress the dye π-stacked aggregation on the TiO_2_ surface. The alkyl chains effectively suppress electron recombination between electrons in the conduction band of TiO_2_ and electrolyte, resulting in higher open-circuit voltage and short-circuit current. Such materials having a fluorene substituent group are already used in organic photoconductor (OPC) drums, such as charge transport materials (CTM), and are essential to copier and printer technology. Therefore, due to their high stability and low toxicity, these dyes could be used in DSCs albeit with a somewhat lower efficiency as compared to best Ru-based dyes.

Previous studies have demonstrated than the onerous synthesis and low charge–carrier mobility of spiro-OMeTAD significantly limit its potential of up-scaling for application on DSCs and perovskite solar cells (PSCs). Therefore, the development of hole-transporting material with higher charge mobility would be also a key approach to achieve higher performance in the solid state solar cell. Carbazole-based derivatives have attracted much attention because of their interesting photochemical properties. Recent interest in the carbazole derivatives has been caused by its good charge-transport function, which can be exploited in the molecular design of new types of HTMs in organic light-emitting diodes (OLEDs) and as electron donor group in organic sensitizers of D-π-A-type in DSCs. Another fascinating advantage is the versatility of the carbazole reactive sites that can be substituted with a wide variety of functional groups, allowing fine-tuning of its optical and electrical properties.

### 3.2. Solution-processed quantum dot solar cells

Materials small enough for the quantum confinement effect to come into play show unique optoelectronic properties different from those of bulk materials such as size-dependent emission and optical absorption bands [113,114]. These properties make QDs attractive in the fields of science and technology. Colloidal quantum dots (CQDs) are one of such nanomaterials. They can be dispersed in a solution, and are useful active materials for LEDs [115], solar cells [116], biosensors, and biomarkers [117].

In addition to the unique optical properties originating from the quantum confinement effect, CQDs are compatible with solution-based methods such as spin-coating, dip-coating [118], spray-coating, and microstamping [119].

**Figure 13.**
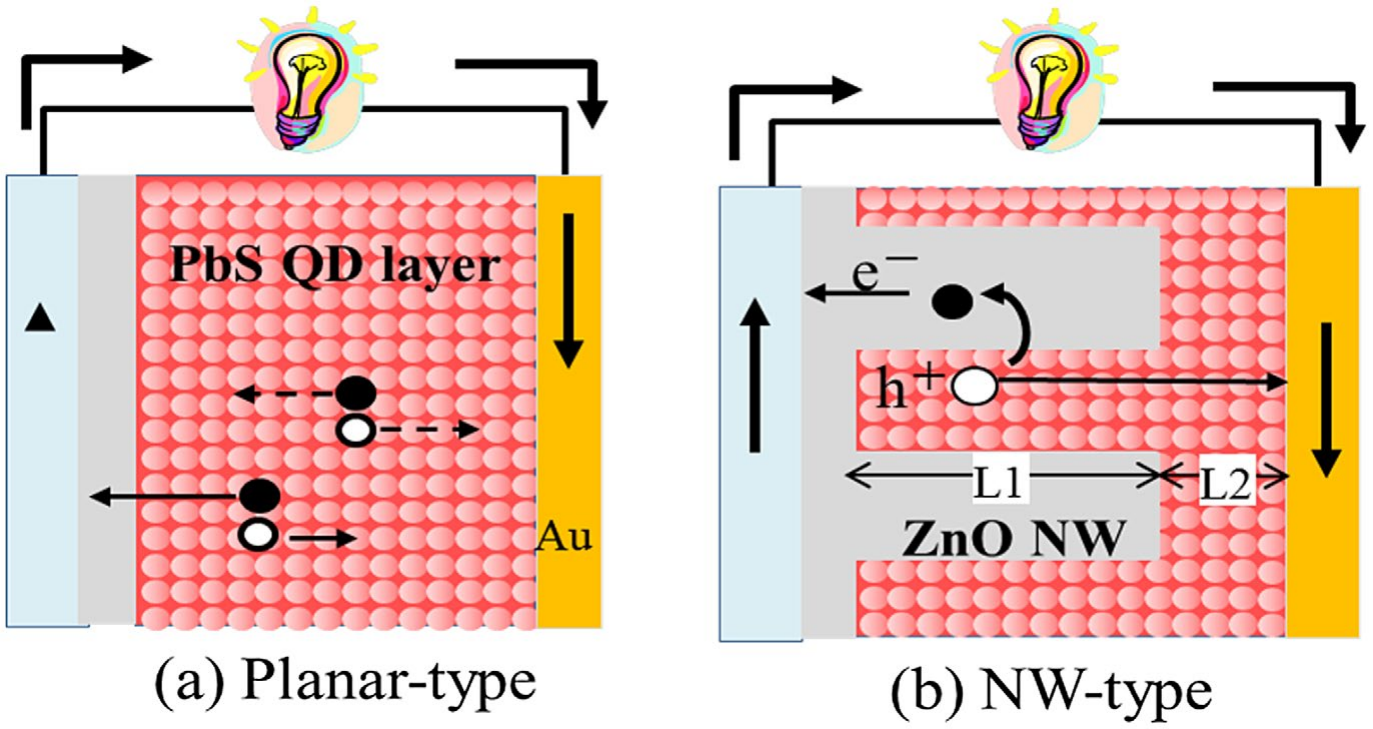
QD-based solar cells of two different types.

Various types of solar cells have been proposed so far [116,120–124]. Among them, interest on Pb-based QD heterojunction solar cells, which are typically formed by depositing the PbS QD layer on top of the flat ZnO layer (referred to herein as planer-type cells) (Figure 13(a)), has rapidly increased these days, and a power conversion efficiency of over 11% has been reported on the solar cells [125,126]. However, the short carrier diffusion lengths of CQD films (having a typical diffusion length is 200–300 nm) makes it difficult to increase power conversion efficiency by thickening CQD active layers. To address this issue, many solar cell structures have been proposed [127–129]. As an example, we will focus in the following on PbS QD-based solar cells with ZnO nanowires (NWs) (referred to as NW-type solar cells) (Figure 13(b)). This solar cell structure allows us to achieve efficient electron transportation as well as high light-harvesting efficiency simultaneously [130–132]. PbS QD solar cells were constructed by combining ZnO NWs with PbS CQDs using a spin-coating method (Figure 13(b)) [132].

The PbS QD/ZnO NW cell structure is denoted by ‘NW-type cell (L1 = *X*, L2 = *Y*)’, where *X* (nm) is the length of ZnO NW and *Y* (nm) is the thickness of the PbS QD overlayer. Planar-type cells were also constructed in a similar way.

**Figure 14.**
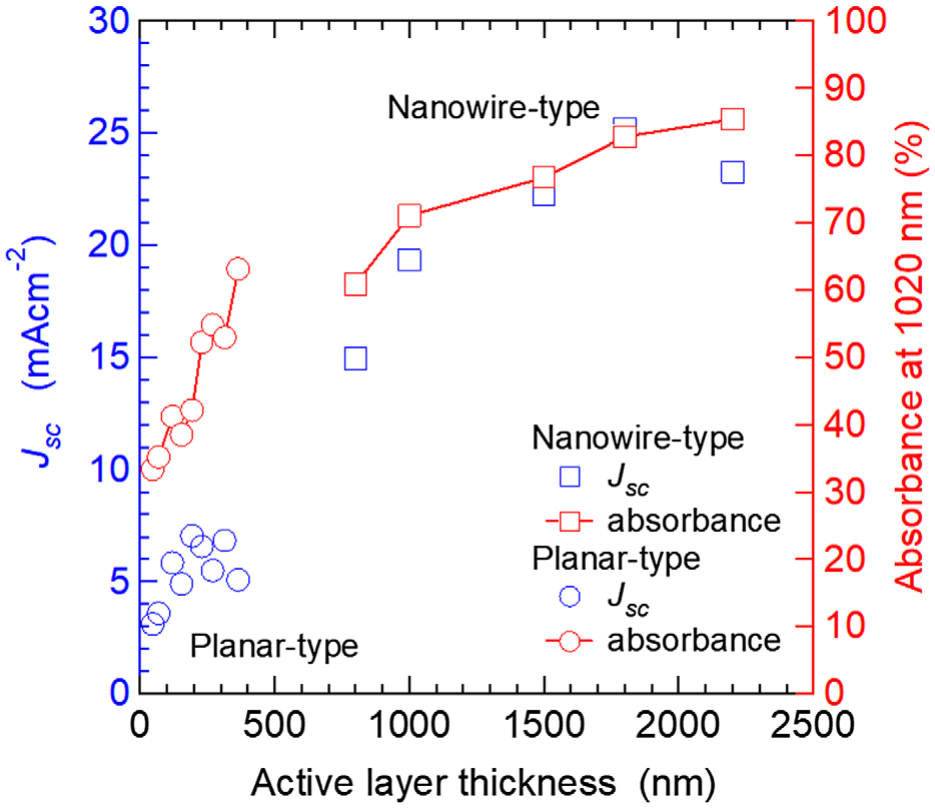
Active layer thickness dependence of *J*_sc_ and absorbance of planar and NW-type solar cells.

The short-circuit current density (*J*_sc_) and absorbance at 1020 nm (@ the first exciton absorption peak) of the two different types of solar cells are plotted as a function of active layer thickness in Figure 14. The *J*_sc_ of the planar-type cells increased until the active layer thickness reached 200 nm owing to the increase in light-harvesting efficiency, and then decreased on further increase in layer thickness. This behavior is mainly attributed to the limitation of carrier diffusion length. In contrast, the *J*_sc_ of NW-type cells (L1 = 400–1500 nm, L2 = 300 nm) increased with active layer thickness (L1 + L2) and reached a maximum at 1800 nm, approximately six times as thick as the typical carrier diffusion length of PbS QD-based cells. Figure 13 shows that electron transport in the PbS QD region is a limiting step in the photoelectron conversion process of PbS QD-based solar cells. Once good electron pathways are established by incorporating ZnO NWs, holes left behind in the PbS QD region can diffuse over 1 μm (Figure 14). We also achieved EQE values of approximately 60% at the first exciton absorption peak (1.02 μm) and over 80% in the visible region by optimizing the morphologies of the ZnO NWs [134,135].

**Figure 15.**
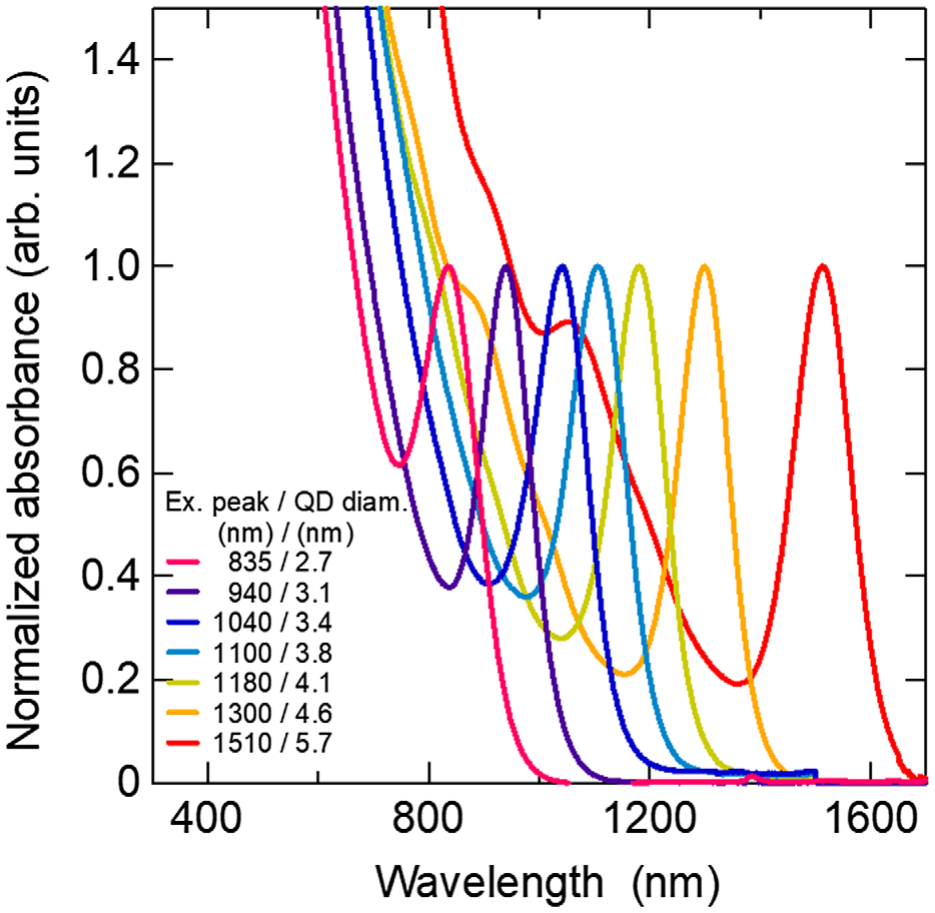
Absorption spectra of PbS CQD solutions. CQD diameters obtained from an empirical equation [133] are shown in the legend along with the first exciton absorption peaks. Absorption spectra are normalized with respect to the exciton peak.

**Figure 16.**
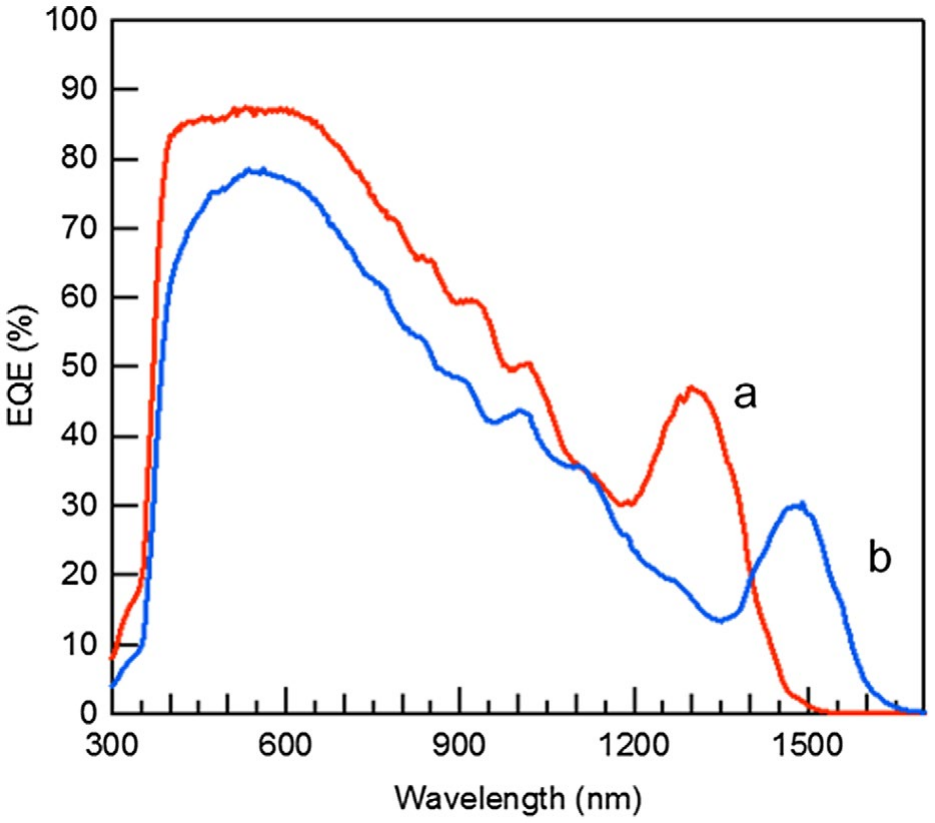
EQE spectra of PbS QD/ZnO NW solar cells. The solar cells (a) and (b) give the exciton peak at 1300 and 1500 nm, respectively.

The bandgap of bulk PbS is located at approximately 3.1 μm (0.4 eV) and can be tuned from the visible to the short-wavelength infrared by adjusting quantum dot sizes. This property makes PbS CQDs attractive materials for the middle and/or bottom subcells of multijunction solar cells [136,137]. To confirm the usefulness of PbS CQD as photoactive materials for multijunction solar cells. PbS CQDs giving different first exciton absorption peaks were synthesized by following a method reported in Ref. [138] (Figure 15). NW-type solar cells were constructed by a layer-by-layer deposition method. The EQE spectra of the NW-type solar cells give the EQE peaks originating from the first exciton absorption. From the EQE spectra, we confirmed that the solar cells can convert photon energy to electricity in a wide range of the solar spectrum (Figure 16). In the solar cell (a), the EQE originating from the 1300-nm-exciton absorption peak reaches 47%, which is, to the best of our knowledge, the record value for PbS QD solar cells. While the solar cell (b) gives a power conversion efficiency of 5.5%, which is, to the best of our knowledge, the highest efficiency ever reported on the solution processed solar cells whose EQE onset is located in the short-wave infrared (SWIR) region.

**Figure 17.**
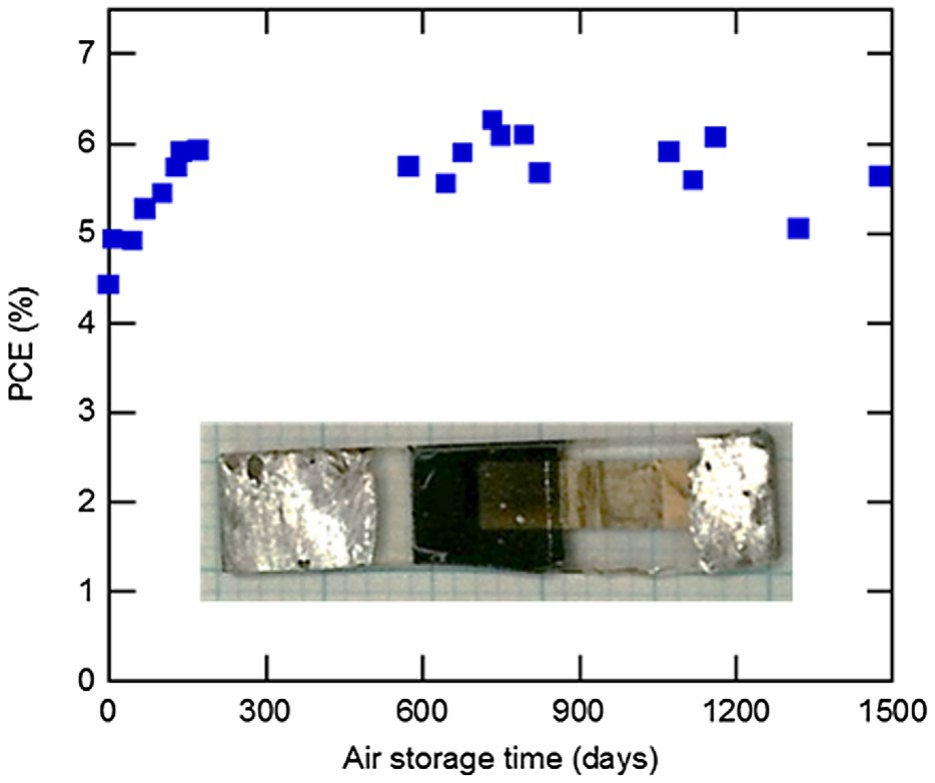
Air-stability tests performed on NW-type solar cells.

Long-term stability is one of the most important properties in practical applications. We constructed NW-type solar cells using the 1.2–μm-thick nanowire layer infiltrated with PbS QDs bearing Br ligands (the inset figure of Figure 17). The solar cells were verified to achieve not less than 4-year air stability, and show no noticeable degradation (Figure 17) [139]. Continuous light soaking tests were also carried out on the solar cells using a solar simulator (AM1.5G 100 mW cm^−2^). The power conversion efficiency was found to keep approximately 90% of the initial value at the end of the 3000-h light soaking test [140]. We have been developing PbS QD/ZnO NW solar cells (NW-type solar cells) that include spatially separated pathways for electrons and holes. The PbS QD/ZnO NW hybrid structures allow us to increase light harvesting efficiency and carrier collection efficiency simultaneously. Based on this strategy, we succeeded in extending an EQE onset wavelength to the SWIR region. PbS QD/ZnO NW solar cells were verified to convert solar energy to electricity in a wide range of solar spectrum from 300 nm to approximately 2000 nm. Moreover, PbS CQDs are compatible with solution-based solar cell technology. These characteristic features show that PbS colloidal quantum dots are one of the most promising photovoltaic materials not only for single-junction solar cells but also for the middle and/or bottom cells of multijunction solar cells. Besides Pb- and Cd-based QDs, there are a wide range of options of CQDs [130,131]. Finally, to develop eco-friendly quantum dot solar cells CuInS_2_ and AgSiS_2_ can be used [141,142].

### 3.3. Organic photovoltaics (OPV)

Organic photovoltaics is part of the so-called third generation of photovoltaic panels together with dye-sensitized solar cells (DSSC), hybrid perovskite solar cells (hPSC), and quantum-dots solar cells (QDSC). Since the introduction of the bulk-heterojunction concept in 1995 [143], the continuous development of novel chemical structures led to the increase of the power conversion efficiency (PCE) up to 13% for single-junction cell [144]. While still less efficient than the other solar cell technologies, organic photovoltaics has advantages which make them interesting for specific applications. The flexibility and lightweight make them useful for nomad applications, while the possibility to tune the color, the shape, and the transparency open the route of the integration in modern and esthetic features [145]. Additionally, organic photovoltaics panels have a low-energy pay-back time [146], low carbon print, [147] and are also efficient with low light intensity (for indoor applications) [148].

#### 3.3.1. Non-fullerene acceptors (NFA)

Since the development of the bulk heterojunction concept [143], the most commonly used acceptor materials in OPV devices are fullerenes and their derivatives. [6,6]-phenyl-C_61_-butyric acid methyl ester (PC_60_BM) has been for 15 years the benchmark for the acceptor material [149] while other derivatives such as [6,6]-phenyl-C_71_-butyric acid methyl ester (PC_70_BM) [150,151] or indene-C_60_ bisadduct (IC_60_BA) [152], were used to improve the absorption in the visible region and the open-circuit voltage, respectively. High efficiencies, up to 11.7%, have been reached with such acceptor [153]. However, these materials present several drawbacks: their poor absorption in the visible region [150], the important energy losses [154] and the instability they induce in the bulk heterojunction [155]. Recent advances have led to the development of alternative acceptor materials for OPV devices. Holliday et al. developed small molecules based on indacenodithiophene core flanked with benzothiadiazole and rhodanine groups (IDTBR) which allowed to overcome the limitation of P3HT:PCBM-based solar cells [156,157]. Indeed, such acceptor contributes efficiently to the photocurrent generation as it presents a complementary absorption to P3HT and boosts the open-circuit voltage due to lower energy losses. As a consequence, P3HT:IDTBR solar cells reached 6.4% power conversion efficiency [157]. These authors also showed that such acceptor can be efficient with a low bandgap donor polymer PffBT4T-2DT. Using IDTBR instead of PCBM allows to reduce the energy losses in the device. Open-circuit voltage above 1 V was achieved and power conversion efficiencies close to 10% were reached, compared to 7.5% with PCBM [158]. Similarly another n-type organic semiconductor, defined as ITIC, presented state-of-the art performances (PCE = 11%) in combination with PBDB-T, a donor polymer semiconductor [159]. The enhancement of the performances in comparison with devices made with PCBM (PCE = 7.5%) is mainly attributed to the additional charge generation due to the complementary absorption of both organic semiconductors. N-type polymeric semiconductor has also seen a strong development in the last years [160]. Perylene diimide and nanphthalene diimide containing polymers have been developed and optimized to replace PCBM. Among them nanphthalene diimide bithiophene also known as N2200 has allowed the fabrication of all-polymer solar cells with PCE of 8.27% due to complementary absorption between N2200 as acceptor and benzodithiophene-alt-benzo-triazole, a medium bandgap copolymer, as donor [161]. In summary, a lot of efforts have been made to find a suitable replacement for fullerene derivatives. Photovoltaic devices made with non-fullerene acceptors reach state-of-the art performances. But more interestingly, due to their contribution in the charge generation and the lower energy loss, such new n-type organic semiconductor opens the route to organic photovoltaics with very high performances. A recent report already published a record power conversion efficiency of 13% with non-fullerene acceptor [144].

#### 3.3.2. Stability improvement

In order to turn the organic photovoltaic into a mature technology, the high performances must remain stable over time. It is commonly accepted that the aging of an OPV device can be divided into 3 steps: (1) a rapid loss of efficiency known as the burn-in, (2) a linear loss of long duration, and (3) an eventual catastrophic failure [162]. The causes of the different losses are multiple and depend not only on the architecture of the device but also on the nature of the organic semiconductor used. The development of the inverted architecture in 2006 has been an important step forward in order to design long-lived solar cells [163–165]. It resulted in air-stable devices and allowed to perform the fabrication process in air. The burn-in is a major issue in organic photovoltaics as it can lead to 20–60% loss of the initial performances [166–168]. Recently, it has been proposed to classify the burn-in period following two phenomena: a loss of short-circuit current (*J*_sc_) or a loss of open-circuit voltage (*V*_oc_). Heumueller et al. have investigated the burn-in involving a loss of *J*_sc_ [169]. They attributed this degradation to the dimerization of the fullerene derivative. Interestingly, they show that the dimerization rate, and, as a consequence the *J*_sc_ burn-in, depends on the active layer morphology. Active layers with well-defined non-crystalline fullerene domains present the highest rate of dimerization and the stronger burn-in. Concerning the burn-in involving of loss of *V*_oc_, it has been proposed that such phenomenon is the consequence of an increase of the energetic disorder in the donor polymer, leading to a redistribution of charges in a broader density of states [170]. Crystalline polymers, having higher charge carrier densities, are less sensitive to increased energetic disorder, thereby reducing the consequences on the *V*_oc_ of such device [171]. The fullerene derivative PCBM has also been identified as another cause of performance loss. Indeed, this small molecule has the tendency to crystallize along aging, modifying therefore the fine morphology of the active layer [155]. Several routes have been identified to freeze the active layer morphology. Derue et al. developed an additive to crosslink the fullerene derivatives and suppress the phase segregation phenomenon for several donor–acceptor blends [172]. To summarize, a lot of progress have been made in the identification of the degradation mechanisms in OPV devices. The fullerene derivatives are a cause of several degradation routes (long-term crystallization, dimerization…) and the development of efficient non-fullerene acceptors can be a solution to those issues. Several report already show that devices with NFA present better stability than those based on fullerene derivatives [173,174].

#### 3.3.3. Toxicity

**Figure 18.**
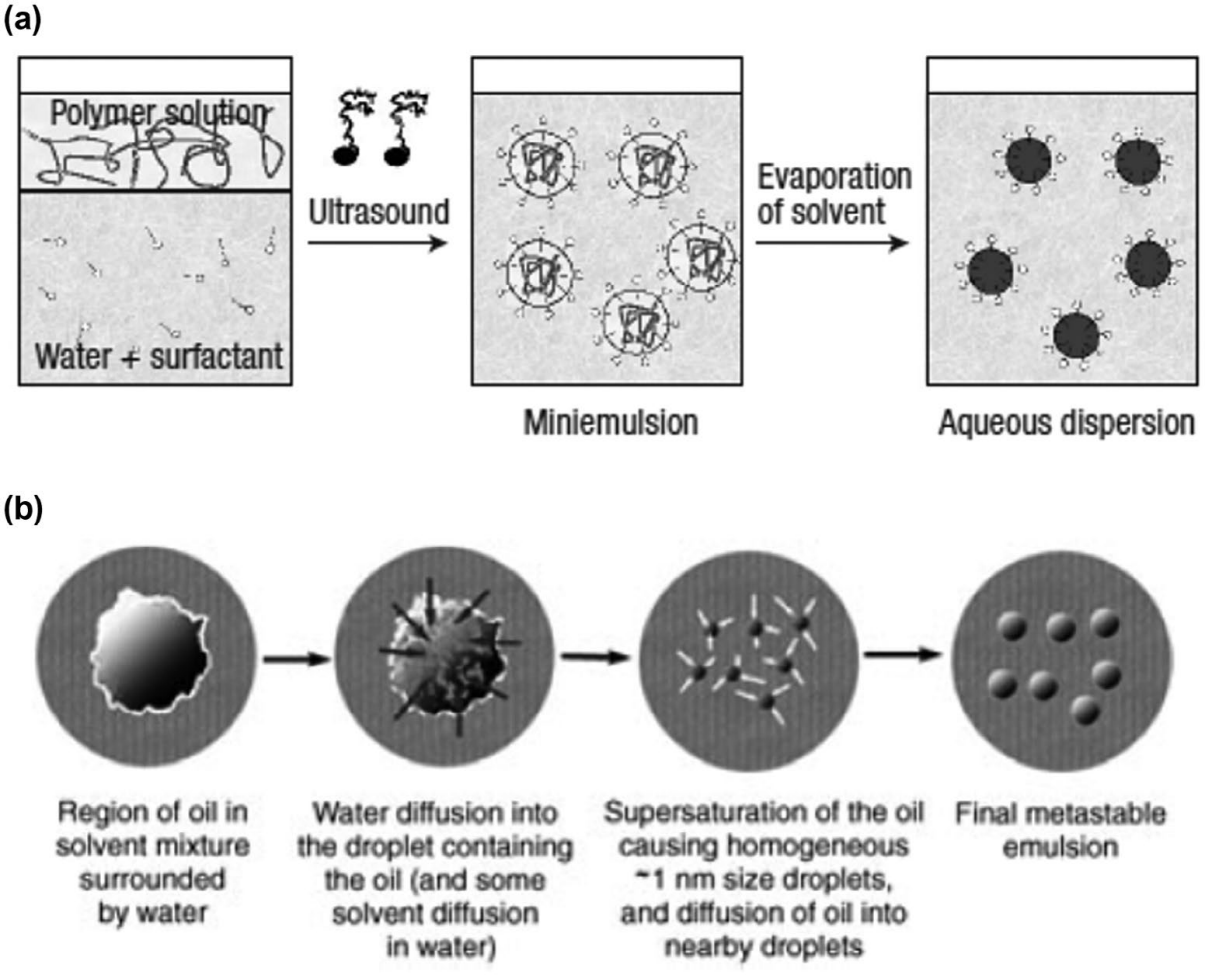
(a) Mini-emulsion technique to formulate aqueous-based organic semiconductor inks reproduced with permission from [178]. (b) Mechanism of nanoparticle generation and growth in the case of nanoprecipitation method. Reproduced with permission from [188].

Another point to be addressed in order to send this technology a step further is the replacement of the commonly used toxic solvent. Indeed, most of the high-efficiency OPV devices were obtained using chlorinated and/or aromatic solvents. The substitution of such solvents with less toxic ones, such as xylene [175,176], while compatible with the industry, has still a non-negligible impact on the environment and working conditions. Eco-friendly processes are under investigation and the development of nanoparticulate organic photovoltaics (NPOPV) is one of them. It began with the work of Thomas Kietzke and Katharina Landfester in 2002 [177–179]. They applied the mini-emulsion technique to generate organic semiconductors nanoparticles dispersed in an aqueous medium with poly(9,9-dioctylfluorene-co-N,N-bis(4-butylphenyl)-N,N-diphenyl-1,4-phenylenediamine) (PFB) as the donor material and poly(9,9-dioctylfluorene-co-benzothiadiazole (F8BT) as the acceptor one. This technique implies the use of two non-miscible solvents (chloroform and water) and a surfactant (Figure 18(a)). Even though the efficiency was low (4% IPCE), those articles were the first proofs of concept for the fabrication of organic solar cells with water-based inks. For several years the efficiencies of OPV devices prepared with water-based inks remained very low, below 1% [180,181], but in 2013, 2 and 2.5% were achieved with P3HT:PCBM [182] and P3HT:ICBA [183] donor–acceptor active layer. They identified a core-shell morphology, with PCBM-rich core and P3HT-rich shell in accordance with the Flory–Huggins free energy of mixing theory [183,184]. Since then, several other polymer donor materials were used to improve the efficiency, a copolymer based on thiophene and quinoxaline units (TQ1) [185] and a low bandgap copolymer (PBDTTPD) which gives 7% average PCE in standard condition (deposited from chlorinated solvents) [186]. Efficiencies up to 3.8% were achieved using such water-based inks [187].

Another method can be used to prepare colloidal inks: the nanoprecipitation. Such technique, based on the supersaturation principle, leads in some specific conditions to the spontaneous generation of nanoparticles dispersed in the anti-solvent [188]. Contrary to the mini-emulsion technique, the solvent and the anti-solvent have to be miscible. The organic semiconductors dissolved in the solvent are injected in the anti-solvent. The materials, which solubility decreases in the solvent mixture, precipitate first and form nanoparticles (generation step), which then percolate (growth step), until they reach a critical diameter which allow them to remain stable in the medium (Figure 18(b)) [188]. Compared to the mini-emulsion technique, such method allows the formulation of surfactant-free dispersions. Using such procedure, Gärtner et al. generated P3HT:ICBA NP dispersion in ethanol and methanol. They fabricated P3HT:ICBA-based organic solar cells and obtained a record PCE of 4% using an optimized thermal annealing step to induce the coalescence of the NP and the formation of a homogeneous film [189,190].

#### 3.3.4. Perspectives

This literature survey on OPV showed that the recent advances allow nowadays the fabrication of devices with power conversion efficiencies around 10% for many donor–acceptor systems. The development of novel n-type organic semiconductors is very promising as it can be a solution to overcome the limitation of devices made with fullerene derivatives and reach even higher power conversion efficiency. The stability issue has been investigated deeply and some strategies have been developed to eliminate or mitigate some degradation mechanisms (phase-segregation, dimerization…).

The replacement of aromatic toxic solvent by water or alcohol to develop eco-friendly processes is still at the first stage and most of the devices were fabricated with P3HT as donor polymer. In order to improve the efficiency of NPOPV devices, it will be important in the future to shift to efficient donor–acceptor systems using low bandgap donor polymer and non-fullerene acceptors. Additionally, the PCEs reached with NPOPV devices are still a step lower (30–40%) than those obtained with chlorinated and/or aromatic solvents. These lower performances are due to non-optimized morphology. Indeed, most of the nanoparticles generated are donor–acceptor mixtures with uncontrolled domain sizes. There are some solutions to overcome those limitations. The size of the NP can be tuned by modification of the mixing conditions (ratio solvent/anti-solvent, concentration) [191], or using microfluidic systems and supercritical anti-solvent (SAS) [192]. The morphology of the NP can be controlled using the solvent selectivity, which allows the fabrication of well-defined core-shell donor–acceptor NP [193].

The combination of those different strategies (efficient donor–acceptor systems, control of the size, and the morphology) can be a solution to develop highly efficient OPV devices using eco-friendly processes.

### 3.4. Perovskite solar cells

**Figure 19.**
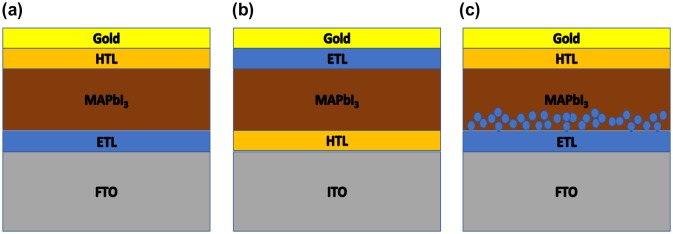
Main PSCs architectures. (a) The planar structure with the electron transport layer (ETL) connected to the front surface electrode and the hole transport layer (HTL) connected to the rear surface electrode. (b) The inverted structure with HTL connected to the front surface electrode and ETL connected to the rear surface electrode. (c) The mesoscopic architecture with a mesoporous layer at the interface between the ETL and the perovskite layer.

Perovskite solar cells (PSCs) have recently attracted great attention due to the large diversity of low-cost processes existing to produce them, the versatility of structure and materials that can be used (see Figure 19) and the excellent opto-electronic properties (strong absorption and high carrier diffusion length) of perovskite material such as methyl ammonium lead iodide (MAPbI_3_) [103,194–197]. The power conversion efficiency (PCE) of PSCs has dramatically improved to over 20% in a relatively short period [198]. Despite their interesting characteristics (low production cost and high efficiencies), several important challenges remain for their commercialization such as: hysteresis in I–V curves; cells stability; ecotoxicity due to the presence of soluble lead compound in the cells and the scaling up of the spin coating process used to produce high efficiencies PSCs [195,199]. However, recently progresses on these main issues have been made, giving the hope that solar cells including perovskite layers will be soon commercialized [197,200–204]. These progresses have been realized on the three main layers composing PSCs (the perovskite layer, the electron, and the hole transport layers) and on the PSCs architecture, improving their efficiency, stability, and reducing the hysteresis. Moreover, many improvements have been made on tandem structure which could compete with single-junction crystalline silicon solar cells in terms of market throughput. In this section, challenges and recent progresses on PSCs hysteresis reduction, efficiency, processing, and stability will be presented.

#### 3.4.1. Hysteresis reduction

**Figure 20.**
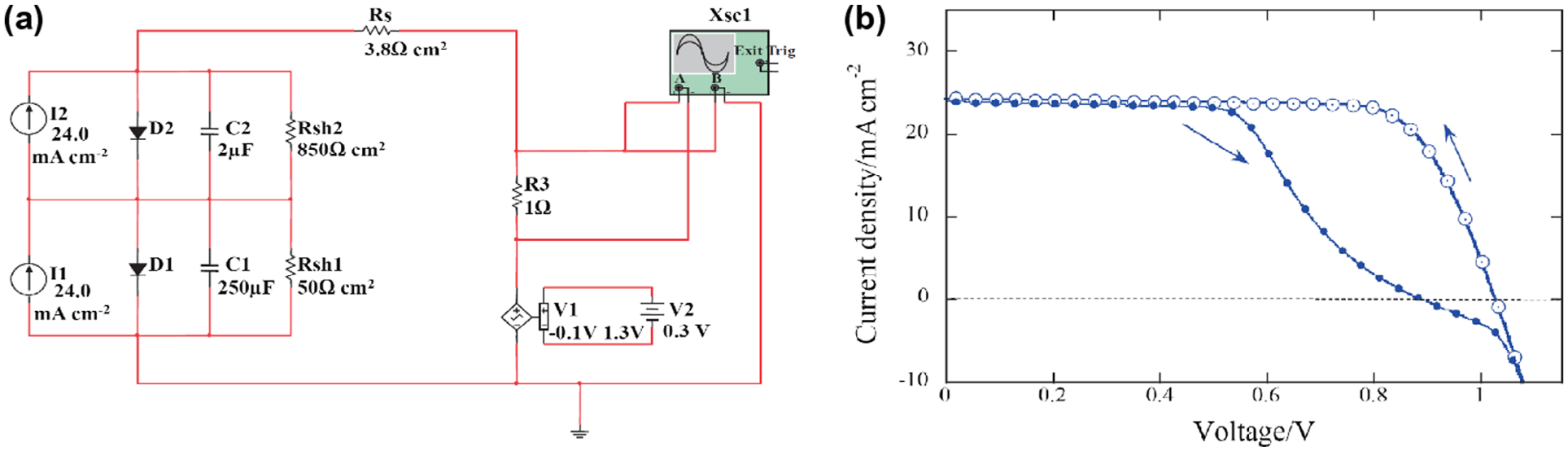
(a) Equivalent circuit model having a capacitance to reproduce the hysteresis phenomenon observed in PSCs. (b) Simulated I–V curve reproducing the large hysteresis observed in PSCs. Reproduced from reference [208] with the permission of Chemistry Letters.

As briefly introduced before, a major problem in PSCs is the hysteretic behavior observed in current density–voltage (J-V) curves [199]. This phenomenon is a notable mismatch between J–V curves measured during a forward scan (from negative to positive voltage) and a backward scan (from positive to negative voltage). The main consequence is the difficulty to determine the PSCs efficiency. Until now, the origin of hysteresis has been mainly discussed as coming from ferroelectric polarization, charge accumulation at the interfaces due to trapping de-trapping, and/or ionic migration of the perovskite [205–207]. We have proposed an equivalent circuit model that includes a capacitance induced by charge accumulations at the interface [208]. This model presented in Figure 20(a) can reproduce the large hysteresis observed in experimental data as it can be seen in Figure 20(b). We are now working on understanding the capacitance origin using the software SILVACO ATLAS [209].

Since the first identification of the hysteresis phenomenon in PSCs in 2014, no direct proof on the mechanism at the origin of the hysteresis has been published. Nevertheless, many strategies have been developed successfully to overcome this detrimental effect. Most of these strategies focus on modifying the interface between the selective contacts and the perovskite layer. Among the strategies developed to suppress the hysteresis, one of the first approaches proposed was to introduce fullerene (C_60_) at the compact TiO_2_/MAPbI_3_ interface in the standard planar structure [210]. Using this approach efficiency above 19% have been recently obtained [204]. Another strategy was to introduce mesoporous TiO_2_ layer at the compact TiO_2_/MAPbI_3_ interface [202]. Using this mesoscopic structure efficiency above 20% has been reached by doping the mp-TiO_2_ with Li [202]. Another successful approach which has been proposed was to use an inverted structure using NiO_x_ and PTAA as the hole and electron transport layer, respectively [203]. With this structure, the hysteresis has been strongly reduced and efficiency over 18% were achieved [203]. As one can notice playing on the PSCs structure and especially on both interface of the perovskite layer has been an efficient way to reduce the hysteresis in PSCs. This has been explained by the passivation of defects and the reduction of charge accumulation at the interfaces [205,207,211].

#### 3.4.2. Efficiency and processing improvement of single-junction and tandem PSCs

**Figure 21.**
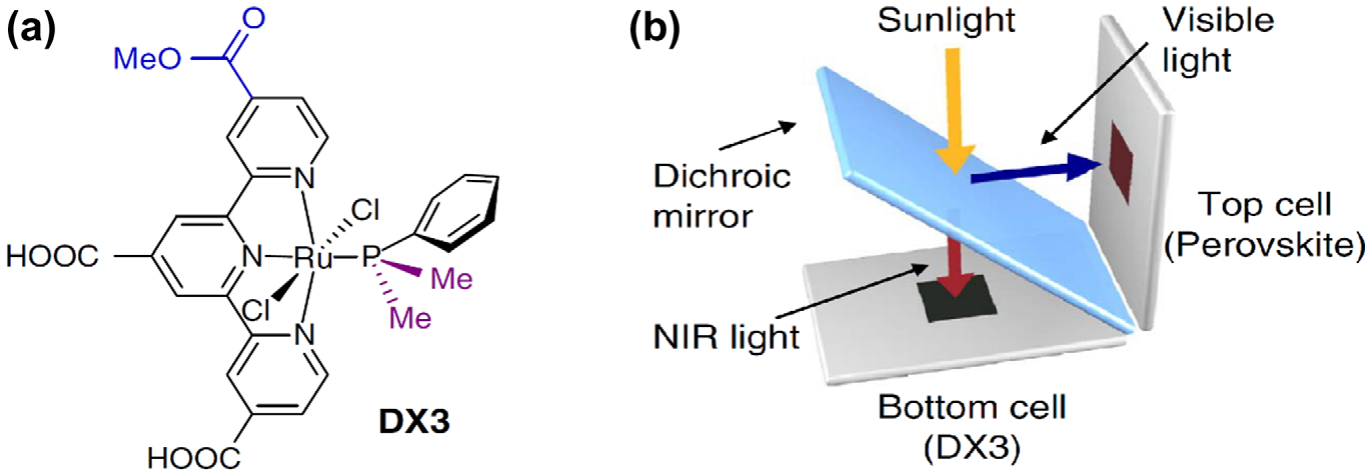
(a) Chemical structure of the panchromatic dye named DX3. (b) Schematic of the DSSC/perovskite tandem solar cell. Adapted from reference [220].

Since the first use of lead halide-based perovskite as a light harvester in Dye-sensitized solar cells (DSCs) different approaches have been used to improve the PSCs optoelectronic properties [194]. One of the first approaches that have led to high-efficiency PSCs was to use a solid type hole transport material such as spiro-OMeTAD [212]. Regarding the work on the interfacial contact, different methods have been developed, such as UV(O_3_)/TiCl_4_ surface treatments, to passivate defects at the compact TiO_2_/MAPbI_3_ interface and improve the efficiency of PSCs [213]. Also, several optimization steps have been performed, notably introducing an anti-solvent step during the MAPbI_3_ spin coating. This approach leads to smoother perovskite layer having larger grain size [214]. Using this method efficiency over 18% has been reached. It has been shown that the perovskite morphology plays an important role on the PSCs efficiency. Larger grain size usually leading to better performance [200,215]. Another strategy was to play on the perovskite composition which allows to tune its energy bandgap, its conduction and valence band energy levels to have a better energy levels alignment with the ETL and the HTL. Using mixed halide perovskite introducing Br into MAPbI_3_ PCE over 20% has been obtained and the device stability has been improved [216]. Recent studies on the Cs_0.05_(MA_0.17_FA_0.83_)_0.95_PbI_0.83_Br_0.17_ compound have shown the great potential of this alloy by achieving efficiency above 20% with a high reproducibility [200]. Since the emergence of PSCs, and thanks to the perovskite large tunable range of bandgap, many groups have started to develop tandem solar cells. Among the main structure developed very interesting results have been achieved using CuInGaSe/perovskite, all perovskite and silicon/perovskite tandem solar cells, with record efficiency reaching 17.8, 20.3, and 23.6%, respectively [217–219]. Also using a panchromatic sensitizer presented in Figure 21(a), coded DX3, that exhibits a broad response into the near-infrared, up to ~1100 nm in DSSC/perovskite tandem solar cells has been developed. A tandem structure presented in Figure 21(b) has been realized achieving a conversion efficiency of 21.5% using a spectral splitting system [220].

These results are reaching record efficiencies gained with single-junction silicon solar cells, but they have been obtained by depositing the perovskite layer via spin coating, which cannot be used for the large-scale production of solar cells. Nevertheless, it is expected that if large-scale process can be used for producing PSCs, perovskite-based tandem solar cells could be soon commercialized. Concerning the large-scale processing of perovskite solar cells, several techniques have been investigated but lead to lower efficiencies. Among them we can notice, spray-coating, blade-coating, roll to roll printing, vapor deposition, and blow-drying [221–225]. This last approach is very promising as it allows to fabricate high-performance devices compared to spray-coating, blade-coating, and roll to roll printing while having lower cost than vapor deposition technique [226].

#### 3.4.3. Stability

**Figure 22.**
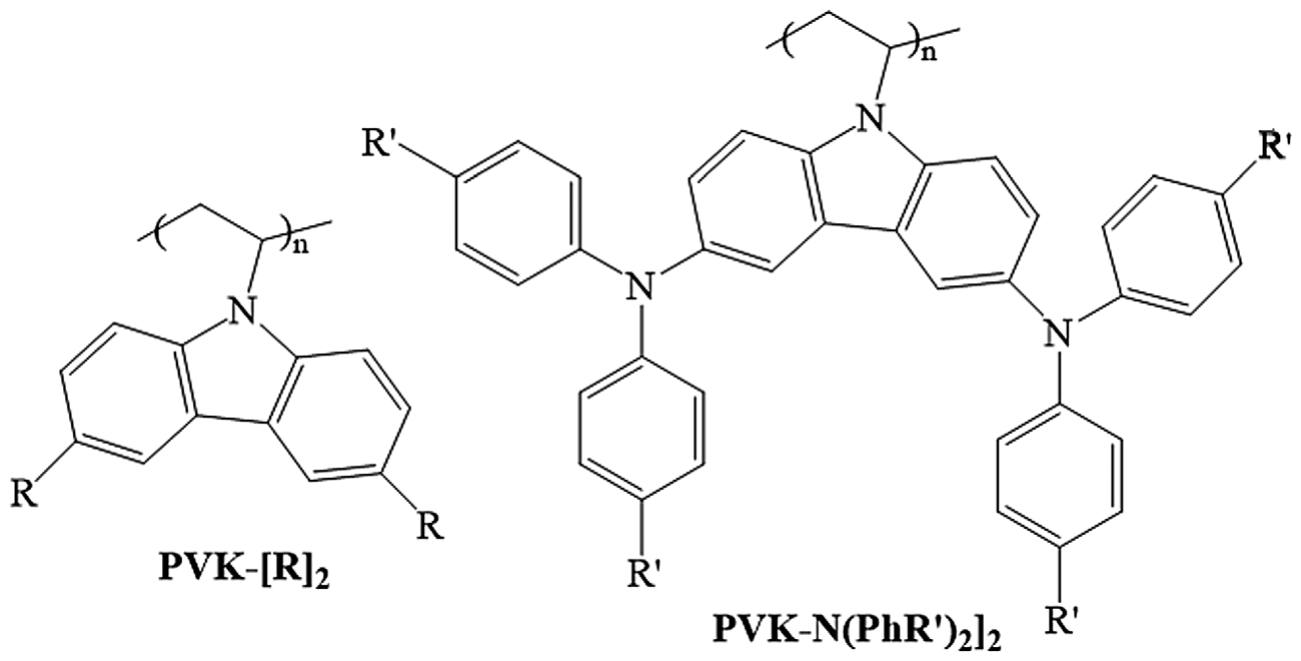
Chemical structure of functionalized poly(vinylcarbazole)s.

Many studies reported that moisture leads to an irreversible degradation of the perovskite layers [227]. This lead to the formation of PbI_2_ which is soluble in water and could thus contaminate the environment, polluting the field and causing eco-toxicological problems. A reduced stability under UV light has been observed and attributed to reactions occurring at the c-TiO_2_ and/or mesoporous TiO_2_ interface with the perovskite layer [228]. One last stability issue is the thermal stability. It is very important to take it into account since during solar cells operation as the temperature can strongly increase. The crystallographic structure being sensitive to temperature, degradation of the active layer can happen after exposing the device at elevated temperatures for medium-long time [229]. To improve the stability of PSCs many strategies have been successfully developed. Structural and compositional engineering have been two efficient ways to dramatically improve the PSCs stability [227]. Concerning the compositional engineering, it has been shown that by modifying the halide and cation composition introducing Br, Cs, and formamidinium in MAPbI_3_, highly stable PSCs could be obtained [200]. Regarding the architecture of the cells, a lot of work has been made on replacing materials such has spiro-OMeTAD due its limited stability [230]. In this context, we specifically designed a series of functionalized poly(vinylcarbazole) bearing alkyl (PVK-[R_2_]_2_) or p-substituted diphenyl amine moieties (PVK-[N(PhR′)_2_]_2_, see Figure 22) [231].

**Figure 23.**
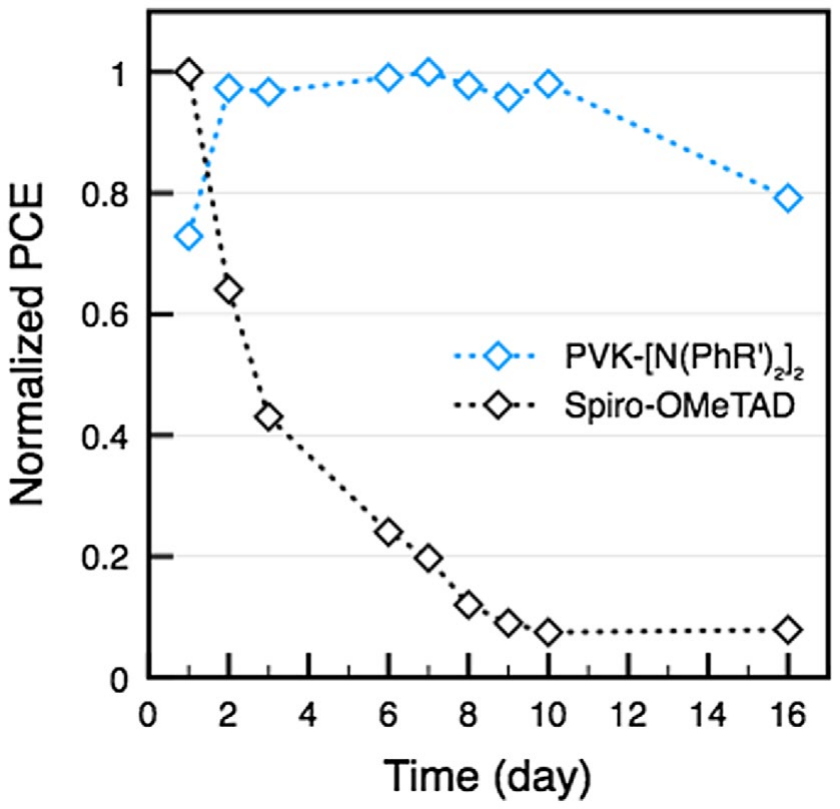
Stability of *spiro*-OMeTAD-based and functionalized PVK-based PSCs stored in dry air without encapsulation.

In a typical SnO_2_:F/c-TiO_2_/CH_3_NH_3_PbI_3−x_Cl_x_/HTM/Au planar PSC, functionalized PVK and spiro-OMeTAD led to similar photovoltaic parameters, i.e. *V*_OC_, *J*_SC,_ and FF, with overall energy conversion efficiencies at 1 sun of about 14%. But a remarkable gain in stability was achieved when functionalized PVK was used as HTM. Efficiency remained stable for more than ten days (under dry-air conditions) rather than one day when using, respectively, functionalized PVK and *spiro*-OMeTAD as HTMs in PSCs (Figure 23). While yet not fully understood such effect could be due to the hydrophobicity of the polymer able to protect the perovskite from atmospheric moisture. Another material reducing the stability of PSCs in classical architecture is TiO_2_ due to its photocatalytic activity [227]. In order to replace TiO_2_ many candidates have been assessed, among them SnO_2_ and BaSnO_3_ are two promising materials for the electron transport layer. Using these materials efficiency above 20% has been achieved with enhanced stability [201,232].

Another strategy that has been used to enhance the PSCs stability was interface engineering. One approach was to introduce ultrathin Al_2_O_3_ layer in the PSCs structure to prevent undesirable chemical reactions. Using this approach several groups have successfully enhanced the stability of PSCs which have remained at 90% of its initial efficiency after storage in air during 24 days [233,234]. Finally, interesting preliminary results have been recently obtained using perovskite layers having a Ruddlesden–Popper structure [235]. Even though the efficiency of these cells is still low (12%) compared to classical architecture, they show promising stability against moisture [235].

#### 3.4.4. Conclusions and outlook

In this section, the main challenges and recent progresses in the development of PSCs have been discussed. It has been shown that interface engineering was an efficient strategy to reduce the hysteresis impeding the efficiency assessment of PSCs. Concerning the efficiency, it has been shown that interface passivation and morphology control through compositional and process engineering was of tremendous importance to produce high-performance perovskite solar cells. Also, different large-scale processing techniques have been presented. Finally, different successful strategies used to improve the PSCs stability have been presented. All these recent developments show that PSCs have a strong potential. They are credible alternative to silicon solar cells for the large-scale development of the solar energy thanks to their promising efficiency and low-cost processing.

## 4. Characterization and modeling

### 4.1. Luminescence-based characterization

The light emitted from the radiative recombination of an electron–hole pair, called luminescence, is a signal that can be detected, and that has been shown to carry numerous material and device properties. Measuring the luminescence is therefore a widely used characterization method. A non-exhaustive list of accessible material properties may include the following: lifetimes, bandgaps, defect position within the bandgap, absorption, carrier temperature (discussed in more details in the Section 2.3), and radiative efficiency. Device-specific quantities can also be determined: series resistances, voltage, saturation currents, and external quantum efficiencies. After briefly reminding some theoretical elements of the luminescence, few examples from the literature illustrating the above list will be given.

The light emission from a semiconductor volume element *ϕ* can be described by the generalized Planck’s law [236]:

(2) $$\phi \left(E\right)=\alpha \left(E\right)\frac{n^{2}}{4\pi^{3}\hbar^{3}c_{0}^{2}}E^{2}\frac{1}{\exp\left(\frac{E-\Delta \mu }{\mathrm{kT}}\right)-1}$$

where *E* is the photon energy, *α* the absorptivity, *n* is the optical index, ħ is the reduced Planck’s constant, *c*_0_ is the speed of light in vacuum, T is the temperature, Δμ is the quasi-Fermi-level splitting. Experimentally, only the light emitted from the material surface can be assessed, so that an integration of equation (2) over the emitting volume, on the light paths leading to surface emission is needed [237–239]. In case the quasi-Fermi-level splitting, the absorption or the temperature is not constant in this volume, the integration of equation (2) is not straightforward. For the sake of simplicity, let us first consider a case in which all those properties are position-independent, so that the surface emission can be written:

(3) $$\Phi_{\mathrm{em}}\left(E\right)=A\left(E\right)\frac{1}{4\pi^{2}\hbar^{3}c_{0}^{2}}E^{2}\frac{1}{\exp\left(\frac{E-\Delta \mu }{\mathrm{kT}}\right)-1}$$

In this equation, the absorptivity *α* (in m^−1^ units) has been replaced by the absorbance *A* (unitless). Although we assumed a constant absorption, quasi-Fermi-level splitting and temperature, we can still consider cases where this is not valid. In those cases, the luminescence can also carry a depth-resolved information, as will be illustrated later.

Equation (3) describes the light emission in a general case, when a semiconductor is brought out of equilibrium by any excitation source. This excitation can be an illumination, so that the luminescence will be termed as photoluminescence, or an applied electrical bias, which is the case of the electroluminescence. In this latter case, a general framework has been developed in terms or reciprocity relations [238,240], which draws a link between the reciprocal mechanisms of a solar cell (conversion of light to electrical energy) and an LED (conversion of electrical energy to light). Considering that phenomena governing carrier transport in the dark and under illumination are similar [238,241–244], and that the light entering and leaving the device follows the same optical path, a reciprocity relation can be derived that relates the external quantum efficiency of a solar cell (i.e. the ratio of collected electrons by incident photons) to the electroluminescence emitted normally from its surface:

(4) $$\Phi_{\text{EL}}\left(E\right)=\text{EQE}\left(E\right)\frac{1}{4\pi^{3}\hbar^{3}c_{0}^{2}}E^{2}\text{exp}\left(\frac{-E}{\mathrm{kT}}\right)\left[\text{exp}\left(\frac{\mathrm{qV}}{\mathrm{kT}}\right)-1\right]$$

Following the same idea, a second reciprocity theorem relates the external quantum efficiency in electroluminescence EQE (i.e. ratio of emitted photons by injected electrons), to the distance of the cell $V_{\text{OC}}$ to the ideal $V_{\text{OC}}^{\text{rad}}$ under the limit of 100% radiative efficiency [238]:

(5) $$\Delta V_{\text{OC}}=V_{\text{OC}}^{\text{rad}}-V_{\text{OC}}=-\frac{\mathrm{kT}}{q}\text{ln}\;\left({\text{EQE}}_{\text{LED}}\right)$$

The set of equations from (2) to (5) provides us with a toolbox, which can be used to access, from the luminescence, the material, and device properties listed in the introduction of this section.

The quasi-Fermi-level splitting, governing the luminescence intensity (equation (2)), is related to the excess carrier population that a semiconductor can maintain. This is therefore a good indicator of the material/device quality, as reflected by the second reciprocity relation (5). Therefore, the luminescence efficiency is considered as a benchmark for comparing different solar cell technologies [238,245–248].

The quasi-Fermi-level splitting, related to the cell voltage, can be obtained by photoluminescence, i.e. without electrical contacts. This has been used to investigate cells at different stages of the fabrication process [249–252]. Combined with the possibility of recording the luminescence with a spatial resolution, inhomogeneous behaviors have been investigated. This can be related to intrinsic (multicrystalline materials) or extrinsic properties (defects and cracks). Examples can be found for multiple technologies, such as multicrystalline silicon [250,253,254], CIGS [255–260], perovskites [260–262], III–V [263,264], dye-sensitized solar cells [265], or multijunction [266]. We can also note a particularly relevant use of the luminescence spectral information, with an absolute calibration, in the case of multijunction devices in the 2-wire configuration. In this geometry, the different subcells are connected in series, so that the voltage delivered by each of them is challenging to access. Nevertheless, since they absorb light in different spectral ranges, their luminescence emissions can be distinguished, from which the subcell voltages are accessed [267–273].

By combining electrical and light excitations of a semiconductor, the emitted luminescence is subject to carrier transport, allowing its properties to be accessed. Series resistances [274–276], and collection efficiency mapping have been reported [239,277–280]. Using the reciprocity relation (4), the EQE could be determined from luminescence [281–283]. Interestingly, close to the bandgap, the current generated by a light excitation is low, so that the signal to noise ratio for the direct measurement of the EQE is low. By contrast, the light emission is the strongest in this energy range, so that the EQE determined by luminescence is more precise. Oppositely, for energies higher than the bandgap, no luminescence can be recorded, but the generated electrical current is stronger. Therefore, improved EQE results can be obtained by combining electrical measurements for energies higher than the bandgap, and luminescence measurements for energies close to the bandgap.

The absorption, entering the luminescence equation (1), illustrates that electron and hole states are coupled radiatively. Thanks to this phenomenon, information on the density of states (conduction and valence bands, dopant, defects, excitons) can be investigated [248,284,285].

In the derivation of the light emitted from the surface, we assumed a constant quasi-Fermi-level splitting, absorption and temperature, for the sake of simplicity. However, this may not be verified, e.g. due to diffusion length in the order of the sample thickness, or absorption properties variations (such as bandgap grading in CIGS [286], or MQW shape grading during growth [287,288]). This can be used to convert a wavelength resolution into a depth-resolved information; in the same manner as an EQE can carry information on surface recombination or diffusion length [289–291]. Luminescence wavelength resolution can be achieved with a spectrometer [288,292], or with two detectors of different wavelength response [282,293,294] (e.g. with cameras made of different materials, or a camera with a set of filters).

### 4.2. Accurate chemical characterization

In parts of the research community, there is growing recognition that studies and published reports on the properties and behaviors of nanomaterials and cutting-edge architectures often have reported inadequate or incomplete characterization. With the increasing importance of complex and miniaturized system in fundamental research for photovoltaic technological applications (heterostructures, tandems systems, quantum dots…) new characterization requirements emerge, such as fine depth probing to analyze buried interfaces or high spatial resolution, to address multimaterials or textured-based structures. Characterization platforms and strategies combining advanced analytical resources are widely developed with the objective to get a better understanding of the surfaces and interfaces properties and their roles in meeting some of the current challenges and concerns. In addition, it is desirable to recognize the nature of unexpected or underestimated challenges associated with reproducible synthesis and characterization of materials, including the difficulties of maintaining desired materials properties during handling and processing due to their dynamic nature. From characterization point of view, the question of the preservation and the reliability of the original information during material transfer or analysis need also to be carefully considered. Chemical characterizations of solar materials are a key step to improve the fundamental knowledge of physicochemical processes and constitute a robust base to optimize modules elaboration and to reach ultimate solar cells performance. The use of surface sensitive analysis methods (including scanning probe microscopy, X-ray photoelectron, and Auger electron spectroscopies combined with appropriate sputtering conditions) can provide crucial information to photovoltaic issues.

**Figure 24.**
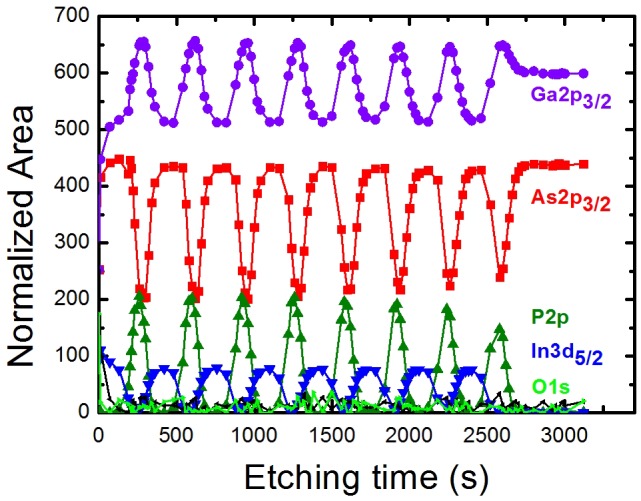
Time evolution during monoatomic Ar^+^ etching of the XPS area of the peaks related to the elements constituting of the InGaAs/GaAsP multilayer stack.

It is widely recognized that as complex systems are developed, surfaces and interfaces begin to dominate and control the properties of nano-structured and multilayered materials. However, the chemical and physical nature of these surfaces and interfaces are often not rigorously measured or reported. Surface analysis techniques need to be extensively used to characterize significant fraction of atoms or molecules associated with surfaces and interfaces, like impurities, surface contamination, surface enrichment, or depletion which can dominate material properties. The combination of X-ray photoelectron spectroscopy (XPS), Auger electron spectroscopy (AES), and low-energy ion spectroscopy with a respective escape depth of less than 10, 4, and 1 nm is of particular interest. A particular case is presented herein on the highly efficient III–V multijunction cells, for which the capacity to investigate the surface and interface phenomena is particularly crucial to precisely optimize each step of the global cell elaboration process. This is especially true in heterostructures with multiple quantum wells, where the MQW are used to adjust the absorption edge keeping the lattice strain balanced [295]. Figure 24 shows that such achievement is possible by XPS Ar^+^ depth profile on III–V multilayer stacks of InGaAs/GaAsP, where each layer is about 10-nm thick (13.5 nm/7.8 nm). The layers appear well separated and characterized with really low levels of oxygen, which could be related to some artifact during etching.

Conventional and time-of-flight secondary ion mass spectrometry (TOFS-SIMS) profiling and imaging techniques bring complementary chemical information to the one obtained by such sequential depth-profiling analysis. Indeed, instead of the abraded surface, the removed matter during abrasion is measured. These mass spectrometry techniques are particularly suitable for organic PV devices characterization [296]. On all materials, they take advantages from the high spatial resolution and detection limit and can be quantitative with the implementation of XPS and nano-Auger techniques, allowing to probe chemical environments. The use of physical erosion is a classical way to perform depth analyses but may likely generate artifacts such as preferential sputtering, surface reconstruction or increased surface reactivity. To confirm the results arising from such a profiling method a common approach consists in the implementation of complementary techniques. Nano-Auger spectroscopy is a good candidate as the inherent high spatial resolution of the technique (12-nm routine spot size) enables the direct characterization of elements depth distribution on cross sections. We have made the demonstration of the capabilities of the new generation Auger nanoprobes on reference Al_0.7_Ga_0.3_As/GaAs superlattices and photovoltaic samples [297]. High spatial resolution can be achieved (≈10 nm), depending on the surface topography, Auger process yield of element considered (element sensitivity), and the global chemical state (superficial contamination or oxidation level) and obviously the acquisition mode employed (point, line, or element mapping). Based on the state of the art of Auger performances and limitations published by Martinez et al. [298], nano-Auger represent a powerful tool for numerous photovoltaic devices characterization and is totally compatible with XPS, SIMS and TOFS-SIMS when localized chemical analyses are needed [299]. Nevertheless, the surface preservation and preparation prior to analysis is a key step, even more than for XPS. Different methodologies can be employed: cross-section polishing (using a chemical polisher or focused ion beam) to reduce the roughness, chemical engineering or physical Ar^+^ abrasion to eliminate or reduce surface superficial oxidation layer and carbon contamination [300]. But, it cannot be excluded once again that such surface preparation methods may perturb the original information. We are currently developing cross-chemical characterization methodologies to demonstrate the representativeness of the information and to reach a multiscale characterization.

An important challenge in the future will be the development of new generation Ar^+^ profiling, with adaptable cluster sizes, enabling to preserve the initial information [301]. Such approach allows to accurately follow the diffusion of particular elements in bulk materials or to access buried interfaces. Proofs of concept have been recently achieved on functional oxides which are particularly known to be modified and damaged by traditional monoatomic etching modes [302–304]. Atomic force microscopy experiments could be performed to monitor the effect of the various bombardments on the remaining surface morphology.

### 4.3. Quantum electronic transport modeling

In the past decades, researchers, including the group at IM2NP, have developed models and corresponding numerical calculations suited to the study of the quantum electron transport in nano-scaled semiconductor devices [305–307]. These tools have been adapted to photonic devices and especially to PV cells [308–310]. They could model an ultrathin solar cell [311,312], at the cost of introducing some approximations, such as the radiative limit. It has been also possible to restrict the investigation to a small active region of the device in order to assume a more realistic approach [313,314]. Generally, such approaches are numerically challenging but will be essential to find new concepts. Quantum phenomena open a wide door to innovation because they can lead to nonlinear and counterintuitive behaviors, and possibly to keys to go beyond the SQ limit.

As an example MQW materials which allow to tailor the optical absorption of the solar cell has been investigated [315]. This approach is efficient thanks to strain-balanced architecture which consist of alternating layers of wells and barriers under compressive and tensile stress. This permits to consider a large number of wells while preventing the formation of dislocations during crystal growth. On the other hand, the use of barriers is a drawback for the collection of the photo-generated carriers and more generally for the electronic transport quality in the MQW. Indeed, since transport is a succession of thermal escape, assisted tunnel escape, and, at best, direct tunneling across a barrier, the average carrier velocity is low (≈10^4^ cm/s) [316]. Finally, the recombination rate is large, and impacts both open-circuit voltage and short-circuit current.

Furthermore, thanks to barriers some minibands can occur. The wave functions of carriers in minibands are Bloch waves, meaning that propagation is efficient. Our theoretical study in InGaAs/GaAs/GaAsP cells sheds light on minibands in which the average velocity of carriers is around 10^7^ cm/s. However, we also show that, without an adapted design, such minibands are inefficient since they connect only a few wells. We show that a graded interlayer thickness allows to largely increase the extraction of carriers by the minibands [317].

More generally quantum engineering based on behaviors such as resonant tunneling, miniband, and phonon-assisted tunneling will be necessary to design new concepts like quantum ratchet in intermediate band solar cell or contact in hot-carrier solar cells. For this quantum engineering modeling is essential.

## 5. Conclusions and outlook

Previous sections have described how emerging materials and technologies may help to push solar energy to the next level. We have seen that the margin of progression of photovoltaic solar energy is still very important in terms of achievable efficiencies: even with the current remarkable advances we are only about half way to reach our goal. Today, multijunctions hold the efficiency records, and are benefiting from progresses made possible by nanotechnologies. These are still quite costly and are a complex stack, but simpler alternatives do exist and may ultimately provide a pathway for competitiveness of highest efficiency devices. Other remarkable progresses are also made in terms of reducing active material usage, here again, the limit is quite far away according to the latest advances of nanotechnologies and nanophotonics. In both cases, more proofs of principle are needed as well as the technologies to make them affordable.

The margin of progression is also very large with new sustainable materials (OPV, DSC, hybrid perovskite) because they use very abundant elements. These materials moreover can be prepared at relatively low temperatures, under atmospheric pressure. They can be printed on many types of substrates and have sometimes esthetic appearance so that new applications and new ways of integration can be proposed. Remaining issues include the long-term stability and possible environmental concerns due to the use of toxic elements (e.g. in some perovskites).

Progress is also observed in materials characterization. To give an example, luminescence-based technologies, which can directly map energy conversion properties (via quasi-Fermi-level splitting) from the microscale to the macroscale (even at the solar farm scale) have seen a strong development recently. Those contribute highly to understanding of material, rapid development of new devices and reliability studies of modules.

So, what is left? One of the next big challenges is related to the variability of the solar source, and its ability to meet the demand minute by minute is often questioned. The complementary answers being developed at the moment range from a better management of the source (and sometimes of the usages) to better and cheaper energy storage capabilities. In this way, solar to hydrogen conversion is a promising direction for solar energy. Storing solar energy into hydrogen via water splitting in electrolyzers is considered [318]. The produced hydrogen can also be transported to compensate geographical fluctuation of the solar resources. Solar cells coupled to electrolyzer are currently an active topic, with recent achievement of world records of solar to hydrogen production, at 24.4% in outdoor conditions (using CPV InGaP/GaAs/Ge cells and proton exchange membrane (PEM) electrolyzers) [319], and 30% under normalized illumination (using an InGaP/GaAs/GaInNAsSb triple junction and PEM electrolyzers) [320]. Here as well, emerging technologies may be leading the way for the undergoing global energy transition.

## Disclosure statement

No potential conflict of interest was reported by the authors.

## Funding

This work was supported by the New Energy and Industrial Technology Development Organization (NEDO, Japan) and the Japan Society for the Promotion of Science (JSPS), the CNRS and RCAST (LIA NextPV).
